# Global motion evoked potentials in autistic and dyslexic children: A cross-syndrome approach

**DOI:** 10.1016/j.cortex.2021.06.018

**Published:** 2021-10

**Authors:** Lisa Toffoli, Gaia Scerif, Margaret J. Snowling, Anthony M. Norcia, Catherine Manning

**Affiliations:** a Department of Developmental Psychology and Socialisation, University of Padua, Padova, Italy; b Department of Experimental Psychology, University of Oxford, Oxford, UK; c Department of Psychology, Stanford University, Stanford, CA, USA; d School of Psychology and Clinical Language Sciences, University of Reading, Reading, UK

**Keywords:** Atypical development, Dorsal stream vulnerability, Motion perception, Evoked potentials, Component decomposition

## Abstract

Atypicalities in psychophysical thresholds for global motion processing have been reported in many neurodevelopmental conditions, including autism and dyslexia. Cross-syndrome comparisons of neural dynamics may help determine whether altered motion processing is a general marker of atypical development or condition-specific. Here, we assessed group differences in N2 peak amplitude (previously proposed as a marker of motion-specific processing) in typically developing (n = 57), autistic (n = 29) and dyslexic children (n = 44) aged 6–14 years, in two global motion tasks. High-density EEG data were collected while children judged the direction of global motion stimuli as quickly and accurately as possible, following a period of random motion. Using a data-driven component decomposition technique, we identified a reliable component that was maximal over occipital electrodes and had an N2-like peak at ~160 msec. We found no group differences in N2 peak amplitude, in either task. However, for both autistic and dyslexic children, there was evidence of atypicalities in later stages of processing that require follow up in future research. Our results suggest that early sensory encoding of motion information is unimpaired in dyslexic and autistic children. Group differences in later processing stages could reflect sustained global motion responses, decision-making, metacognitive processes and/or response generation, which may also distinguish between autistic and dyslexic individuals.

## 1 Introduction

Motion perception plays an important role in the developing visual system, influencing cognitive abilities and actions (Braddick et al., 2003). Global motion processing - which requires the integration of motion signals across the visual field - is one aspect of motion perception that follows a particularly protracted developmental trajectory (e.g., Atkinson et al., 2002; Braddick, 1993; Kiorpes et al., 2012; Langrová et al., 2006; Wattam-Bell et al., 2010). Global motion processing is most commonly assessed using the motion coherence threshold - the minimum proportion of coherently moving dots needed to perceive the overall direction of motion, amidst randomly moving noise dots (Newsome & Paré, 1998). Elevated motion coherence thresholds have been reported in individuals with autism (Van der Hallen et al., 2019) and dyslexia (Benassi et al., 2010). Van der Hallen et al. (2019) analysed 28 studies comparing coherent motion perception in autistic individuals and control participants and found a small mean effect (.33), reflecting reduced sensitivity to coherent motion in the autistic population. Benassi et al. (2010) reported a larger effect in their meta-analysis of 35 studies comparing coherent motion sensitivity in dyslexic individuals and age-matched control participants (*d* = .75), with reduced sensitivity to coherent motion in dyslexic individuals.

Efforts have been made to uncover the reasons behind atypical motion processing among different neurodevelopmental conditions. Motion processing develops more slowly than form processing, which has been taken to reflect a more protracted developmental trajectory for the dorsal stream compared to the ventral stream (Atkinson et al., 2002; Braddick et al., 2002; Kiorpes et al., 2012; Langrová et al., 2006). It has been suggested that the dorsal stream is, as a result, particularly vulnerable to atypical development (i.e., the ‘dorsal-stream hypothesis’, Braddick et al., 2003), potentially explaining elevated motion coherence thresholds in a range of developmental conditions. However, it is also possible that reduced sensitivity to coherent motion might arise for distinct reasons in different neurodevelopmental conditions (see Dakin & Frith, 2005; Manning et al., 2015). Investigating the neural correlates of global motion tasks is one way of addressing whether reduced motion sensitivity is a general marker of atypical development or if it is more condition-specific. In particular, using techniques that are sensitive to the multiple processes contributing to motion processing can elucidate whether atypicalities emerge at similar or distinct stages of processing across atypically developing groups.

Much is known about the neural dynamics of motion processing in neurotypical individuals. Adults exhibit three distinct neural peaks in response to motion onset (typically following a stationary stimulus): a first positivity at around 130 msec after motion onset (P1 or P100), a first negativity at around 160–200 msec (N2 or N200) and a second positivity at around 240 msec (P2 or P200) (Kuba et al., 2007). In order to isolate motion directional mechanisms and avoid confounding effects with the onset of spatial temporal luminance modulation, a period of random motion can be presented before the onset of coherent motion (Niedeggen & Wist, 1998, 1999; Patzwahl & Zanker, 2000). In this case, only the N2, but not the P1, is observable after the coherent motion onset (Niedeggen & Wist, 1999), suggesting that the N2 is motion-specific, whereas P1 is sensitive to luminance contrast (Clifford & Ibbotson, 2003; Heinrich, 2007; Niedeggen & Wist, 1999).

Manning et al. (2019) used this paradigm to measure visual evoked potentials to coherent motion in 6- to 12-year-old typically developing children and adults. They used a data-driven dimensionality reduction technique, Reliable Components Analysis (RCA; Dmochowski et al., 2012; Dmochowski et al., 2014; Dmochowski & Norcia, 2015), which maximises trial-to-trial reliability across participants. Results revealed two main components: one of these (the second most reliable component, ‘component 2’) was maximal over occipital electrodes and resembled standard coherent motion evoked potentials reported in adults (Niedeggen & Wist, 1999; Patzwahl & Zanker, 2000). A negative N2-like peak was observed in children and adults at around ~300 msec but, unlike adults, children also had an initial positive P1-like peak, at around ~200 msec. The other component (the first most reliable component, ‘component 1’) resembled the previously reported centro-parietal positivity (CPP; O'Connell et al., 2012; Kelly & O'Connell, 2013) and behaved as a decision-related variable: it scaled in line with motion coherence and increased as a function of time (Manning et al., 2019). The maximum amplitude of this component increased during childhood and decreased again to a small degree for adults, while the latency of the observed peak was shorter in older children and adults compared to younger children. This study suggested that improvements in coherent motion performance during childhood are accompanied by the maturation of neural activity linked to both early sensory and later decision-related processes.

Despite a large number of behavioural studies in atypically developing groups, still little is known about the neural correlates and temporal dynamics of global motion processing in atypical development. Only a few studies have measured visual evoked potentials to coherent motion in autistic and dyslexic populations. Greimel et al. (2013) presented motion coherence stimuli following a period of random motion and compared responses locked to the onset of coherent motion in autistic (n = 16) and typically developing (n = 12) children and adolescents aged 8–16 years. Their results revealed a reduced N2 peak amplitude in the autistic group compared to the typically developing group, but no differences in latency. To our knowledge, the only study to have measured evoked potentials locked to the onset of coherent motion following a period of random motion in dyslexic participants was conducted by Schulte-Körne et al. (2004). Here, dyslexic children (n = 10) had reduced amplitudes of a positive peak between 300 and 800 msec compared to typically developing children (n = 12) in response to coherent motion, but no information relating to the N2 was reported.

Three further studies assessed evoked potentials to motion coherence onset without an initial period of random motion in dyslexic individuals. Jednoróg et al. (2011) did not find any overall group differences in coherent motion evoked responses, but reported that while control participants (n = 16) had a higher N2 peak for coherent motion than random motion, the N2 peak did not differentiate between the two types of motion in dyslexic participants (n = 16). Taroyan et al. (2011) also reported no significant differences in visual evoked responses between typically developing (n = 10) and dyslexic (n = 9) adolescents. Similarly, Scheuerpflug et al. (2004) found no significant differences in visual evoked potentials between dyslexic (n = 16) and typically developing (n = 15) children.

The literature to date on visual evoked responses to coherent motion onset in autism and dyslexia is scarce and results are not always comparable due to different methodologies used across studies and experimenter degrees of freedom associated with selecting electrodes and time windows of interest. In the present study, we compared visual evoked responses to global motion onset in typically developing, autistic and dyslexic children. In our paradigm, motion coherence onset was preceded by a period of random motion, which allowed us to isolate directional mechanisms. Additionally, we used the data-driven RCA method as in previous studies of typical development (Manning et al., 2019, 2021) to reduce experimenter degrees of freedom and boost the signal-to-noise ratio of evoked activity. This cross-syndrome approach allows an understanding of whether altered motion processing is a general marker of atypical development or if it is condition-specific.

We administered two tasks: a motion coherence task, where a proportion of dots moved coherently amidst randomly moving dots, and a direction integration task where all the dot directions were sampled from a Gaussian distribution and difficulty was manipulated by varying the standard deviation of this distribution. This direction integration task was previously used by Manning et al. (2015; 2017) alongside the standard motion coherence task. Surprisingly, they found enhanced performance in autistic children compared to typically developing children in the direction integration task but not in the motion coherence task. Since the optimal strategy in the direction integration task is to average across the local dot directions, the authors concluded that autistic children showed an enhanced ability to integrate motion information. Conversely, the motion coherence task could be limited by difficulties segregating the motion of the signal dots from that of the noise dots, meaning that previous reports of reduced motion coherence ability in autism could arise from a reduced ability to filter out noise (noise exclusion; see also Zaidel et al., 2015; van de Cruys et al., 2017). Difficulties with noise exclusion have also been proposed in dyslexia (Conlon et al., 2012; Sperling et al., 2005, 2006), although to our knowledge, the direction integration task has not been used previously to investigate dyslexia. We presented similar tasks in the current study, to assess whether neural differences varied according to task demands.

Similar to Manning et al. (2019), we used high-density EEG and identified reliable components locked to the onset of coherent motion. This approach has advantages over more traditional evoked potential methods that average over a single electrode or set of electrodes by making use of all electrodes and increasing signal-to-noise ratio (see Manning et al., 2019). We expected to find two neural components, with our analyses focusing on the second most reliable component (‘component 2’) to be comparable to previous studies assessing coherent motion evoked potentials over occipital electrodes. We were most interested in the N2-like peak, as the N2 has been proposed as a marker of motion-specific processing (Kubová et al., 1995) and the most consistent precedents in EEG research on autism and dyslexia have focused on this component (e.g., Greimel et al., 2013; Jednoróg et al., 2011). We used a mass univariate approach (Groppe et al., 2011) to compare activity across all timepoints. We pre-registered our research questions and hypotheses for the N2 (https://osf.io/7zmhc*)* but did not pre-register *a priori* hypotheses on later components, as the literature in autism and dyslexia has so far mostly focused on early components like the N2. All deviations from the pre-registered procedures and analysis plans are transparently identified.

### 1.1 Research questions

1. *Do autistic children and dyslexic children exhibit a reduced N2-like peak during the motion coherence task compared to typically developing children?*

Greimel et al. (2013) compared the visual evoked responses of autistic and typically developing children using a similar motion coherence task and paradigm to those used in this study. Their results revealed a reduced N2 amplitude in the autistic sample, in particular over occipital electrodes, but no differences in the P1 amplitude, and no differences in latency in either peak. Based on this study, we expected a reduced N2-like peak among our autistic children. However, no previous study reporting on the N2 for coherent motion evoked potentials in dyslexia applied comparable methods to the ones used in the present study. Given that difficulties in coherent motion processing have been reported both in autism and dyslexia (Benassi et al., 2010; Pellicano & Gibson, 2008; Van der Hallen et al., 2019), and that atypicalities of the N2 have already been observed in the dyslexic population (Jednoróg et al., 2011; Kubová et al., 2015), we expected a similar pattern across both groups. Hence, we hypothesized a reduced N2-like peak in our dyslexic children compared to typically developing children. Any difference in results for autistic and dyslexic children would suggest that motion processing alterations are disorder-specific. We had no *a priori* hypotheses regarding N2 latency as, to our knowledge, no previous studies have reported differences in N2 latency in autism or dyslexia using a similar paradigm to ours.

2. *Do autistic and dyslexic children differ from typically developing children in N2-like peak amplitude during a direction integration task?*

To our knowledge, no studies have measured visual evoked responses during a direction integration task in autistic or dyslexic populations. We optimised the current direction integration task for EEG data collection, rather than estimating threshold estimates, but we hypothesised an increased N2-like amplitude in our autistic children based on previous behavioural findings of enhanced performance in autistic compared to typically developing children (Manning et al., 2015, 2017). Drawing a hypothesis for the dyslexia group was more complicated, given that – as far as we know – no previous study has administered a similar direction integration task to this population. If we assume a general impairment to the magnocellular or dorsal-stream in dyslexia, we would expect a reduced N2-like peak in dyslexic compared to typically developing children in this task, as in the motion coherence task. Conversely, if dyslexic individuals have difficulties with motion coherence processing due to difficulties in perceptual strategies, as in noise exclusion (Conlon et al., 2012), we may expect no significant difference in amplitude of the N2-like peak between dyslexic and typically developing children during the direction integration task.

## 2 Methods

### 2.1 Participants

The sample included 57 typically developing children, 29 children with an autism diagnosis and 44 children with a dyslexia diagnosis,^1^ aged 6–14 years (see Table 1 for demographic information). Participants were recruited from local schools, community contacts and invitations to families who participated in previous studies, as part of larger studies assessing perceptual decision-making in autism and dyslexia using Bayesian models (https://osf.io/znyw2 and https://osf.io/enkwm). These larger studies determined the sample of participants tested. In total, we tested 50 children in each of the autism and dyslexia groups and 60 typically developing children, based on Monte Carlo simulations (Schönbrodt & Wagenmakers, 2016) suggesting that 49 participants per group are required on average to detect a moderate effect size of d = .5. The current study included a subset of these participants, following different pre-registered inclusion criteria (e.g., excluding children who did not complete the task with EEG or who had indications of both autism and dyslexia symptoms). EEG data of three children (one from each group) are missing for the direction integration task due to technical difficulties or the child's wish to complete that session without EEG.

**Table 1:** Demographic information.

	TD (n = 57)	Autistics (n = 29)	Dyslexics (n = 44)
Sex	32M 25F	22M 7F	19M 25F
Age	10.50 (2.22)	11.04 (2.57)	11.02 (1.79)
	(6.55–14.98)	(6.54–14.94)	(8.26–14.53)
Verbal IQ	111.49 (9.19)	109.76 (12.59)	100.23 (9.75)
	(95–132)	(85–137)	(82–118)
Performance IQ	112.46 (13.37)	109.55 (14.59)	101.2 (15.22)
	(81–145)	(78–136)	(72–141)
Full IQ	113.53 (10.21)	110.9 (13.21)	100.64 (12.18)
	(89–135)	(84–133)	(79–132)
SCQ	2.56 (2.75)	19.69 (7.52)	4.91 (3.74)
	(0–12)	(4–32)	(0–14)
TOWRE-2 PDE	106.95 (10.91)	107.76 (11.83)	78.57 (6.97)
	(80–135)	(86–132)	(65–99)
WIAT-III spelling	113.3 (16.33)	106.86 (18.9)	79.73 (8.19)
	(84–153)	(68–152)	(59–99)
Composite score	110.12 (12.55)	107.31 (12.88)	79.15 (6.12)
	(89.5–140.5)	(89.5–142)	(63–88.5)
ADOS Total	–	12.17 (5.37)	–
		(4–27)	
ADOS Severity	–	6.83 (2.12)	–
		(2–10)	

Notes. Data are presented as mean (standard deviation) range for typically developing (TD), autistic and dyslexic children. For the direction integration task, data from three participants (one from each group) were missing (TD, n = 56; autistics, n = 28; dyslexics, n = 43). IQ was measured with the Wechsler Abbreviated Scales of Intelligence (WASI-2; Wechsler, 2011). SCQ = Social Communication Questionnaire (Rutter et al., 2003); TOWRE-2 PDE = Test of Word Reading Efficiency - Phonemic Decoding Efficiency subtest (Torgesen et al., 2012); WIAT-III Spelling = Wechsler Individual Achievement Test - Spelling subtest (Wechsler, 2017); ADOS = Autism Diagnostic Observation Schedule (ADOS-2; Lord et al., 2012). The composite score was obtained by averaging together the TOWRE-2 PDE and the WIAT spelling.

For inclusion in the current dataset, children had to have normal or corrected-to-normal visual acuity (measured using a Snellen acuity chart) and verbal and performance IQ scores above 70 (measured using the Wechsler Abbreviated Scales of Intelligence [WASI-2]; Wechsler, 2011). Parents of all children were asked to complete the Social Communication Questionnaire (SCQ; Rutter et al., 2003). Additionally, autistic children were assessed with the Autism Diagnostic Observation Schedule (ADOS-2; Lord et al., 2012), to quantify autism symptoms. Autistic children were included in the dataset only if they met criteria on the SCQ (total score ≥ 15) and/or ADOS (total score ≥ 7; see Manning et al., 2015). The standard scores from the Wechsler Individual Achievement Test (WIAT-III; Wechsler, 2017) spelling subtest and the Test of Word Reading Efficiency (TOWRE-2; Torgesen, et al., 2012) Phonemic Decoding Efficiency (PDE) subtest were averaged to form a literacy composite score. Children in the dyslexia group were included in the dataset only if their composite score was 89 or below (Snowling et al., 2019). Typically developing children and autistic children were included in the dataset only if their composite score was above 89, and typically developing children and dyslexic children were included only if their SCQ score was below 15. Children with both autism and dyslexia diagnoses were excluded from the dataset.

As shown in Table 1, the groups overlapped in the range of scores for both age and IQ. However, the autistic and dyslexic children had a slightly higher mean age than the typically developing children, with the minimum age in the dyslexic children being higher than that in the other groups. The dyslexic children also had lower mean verbal and performance IQ scores than the typically developing and autistic children. Importantly, the data show that the autistic children generally had SCQ scores within the clinical range for autism while the dyslexic group were similar to controls on this measure. In contrast, the dyslexic group was impaired in reading and spelling whereas the autistic children and controls scored in the normal range for their age on those measures.

### 2.2 Apparatus

The experimental task was presented on a Dell Precision M3800 laptop (2048 × 152 pixels, 60 Hz) using MATLAB (Mathworks, MA, USA) and the Psychophysics Toolbox (Brainard, 1997; Kleiner et al., 2007; Pelli, 1997). EEG signals were acquired with a 128-electrode Hydrocel Geodesic Sensor Net connected to Net Amps 300 (Electrical Geodesics Inc., OR, USA), using NetStation 4.5 software. A photodiode attached to the monitor independently checked the timing of stimulus presentation. Children made their responses using a Cedrus RB-540 response box (Cedrus, CA, USA).

### 2.3 Stimuli

**Fig. 1:**
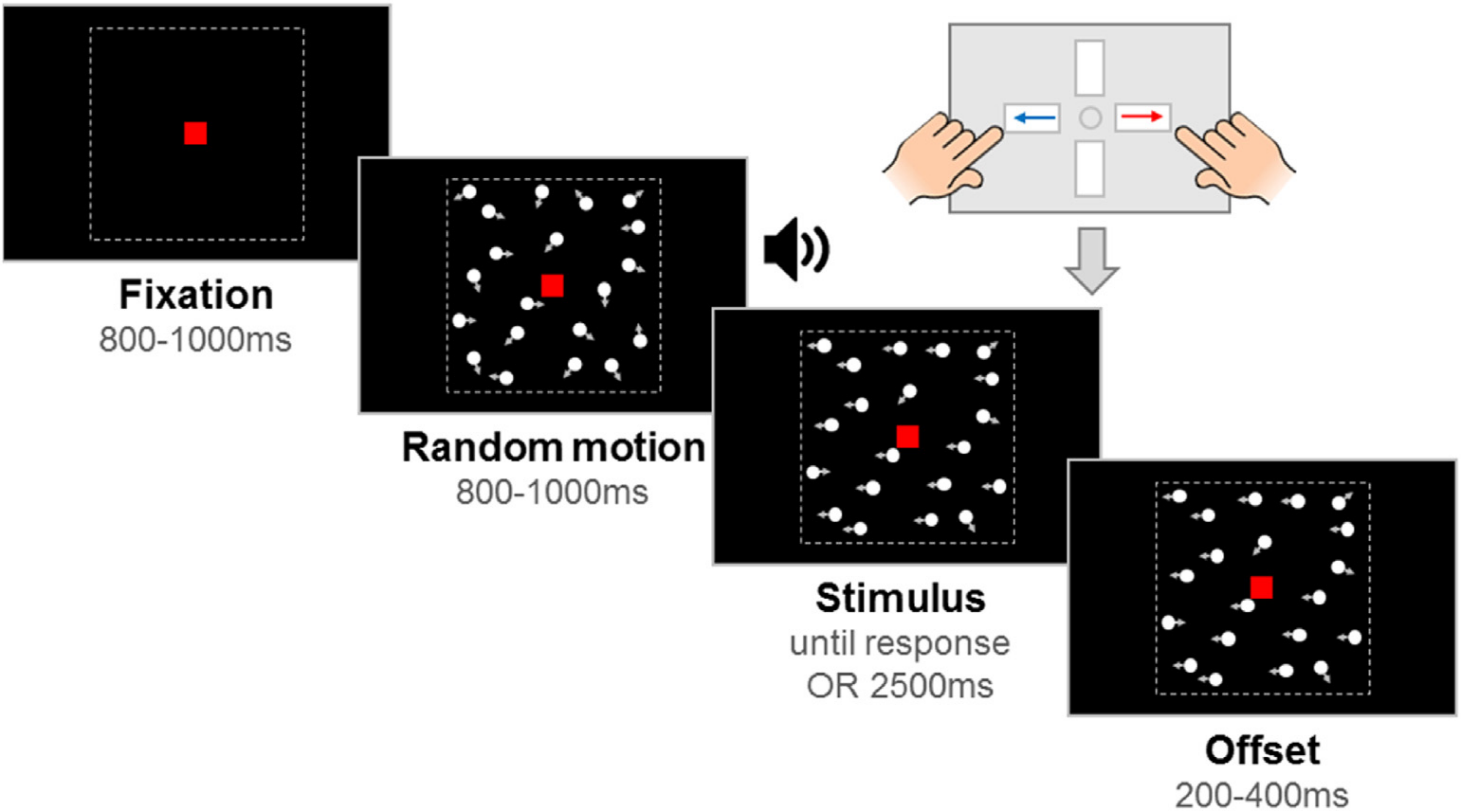
**Schematic representation of trial procedure.** The trial started with an initial *fixation* period that was followed by a *random motion* period consisting of random, incoherent moving dots, which was in turn followed by a stimulus period. The *stimulus* period contained leftward or rightward global motion and the child was required to report the direction using a response box. If there was no response, the stimulus remained on the screen for 2500 msec. The stimulus remained on the screen for an *offset* period after the response or after the maximum stimulus duration was reached. Note that arrows (indicating movement) and dotted lines (marking the square stimulus region) are presented for illustration only. Figure reproduced from https://osf.io/wmtpx/ under a CC-BY4.0 license.

One hundred white stimulus dots (diameter .19°; luminance 248 cs/m^2^), moving at a speed of 6°/s, were randomly positioned within a central square region (10° x 10°) on a black screen (luminance .22 cd/m^2^). The lifetime of the dots was limited to 400 msec (with starting lifetimes being randomised) and dots moving outside the square stimulus region were wrapped around to the opposite side. A red fixation square (.24° x .24°) was present on the screen centre for the entire duration of the trial. Each experimental trial started with a fixation period, followed by a random motion period, a stimulus period and an offset period (see Fig. 1). The fixation period lasted for a randomly selected duration between 800 and 1000 msec, during which only the central fixation square was visible. Stimulus dots first appeared in the random motion period, during which they moved in random directions, for a randomly selected duration between 800 and 1000 msec; this helped us to prevent any confounding effects between the motion onset and the onset of spatial temporal luminance modulations. The start of the stimulus period was signalled with an auditory signal (a short beep), in order to reduce temporal uncertainty. In the stimulus period of the motion coherence task, a proportion of dots moved coherently either leftward or rightward, while the remainder of the dots continued to move randomly. In the stimulus period of the direction integration task, the dot directions were taken from a Gaussian distribution with a mean leftward or rightward direction. The stimulus period ended after the child made a response or after 2500 msec elapsed. Finally, an offset period continued the coherent stimulus presentation for a randomly selected duration between 200 and 400 msec. The jittered durations of the fixation, random motion and offset periods were designed to minimise possible expectancy effects.

### 2.4 Experimental task procedure

Children's motivation was facilitated by presenting the experimental tasks in the context of a child-friendly ‘game’, which was successfully used in a previous study of typically developing children aged 6–12 years (Manning et al., 2019). Participants were invited to play two ‘game’ sessions with 5 ‘levels’ each, in which they had to indicate, as quickly and accurately as possible, the overall direction (left or right) of ‘fireflies’ (white stimulus dots) using a response box. The order in which motion coherence and direction integration tasks – each corresponding to one ‘game’ session – were presented was counterbalanced between participants. We asked children to wait until they heard the auditory tone and then to report their perceived direction of overall motion using a response box, emphasizing that they should be as quick and accurate as possible. The trials in the experimental phase of each task were preceded by an initial combined demonstration, practice and criterion phase (the first ‘level’ for each task).

Children were initially presented with 4 demonstration trials with an unlimited presentation time and no random motion period, so that the experimenter could explain the task. Following this, they were required to pass a criterion of 4 consecutive correct responses within 20 trials for an ‘easy’ stimulus (95% coherence or SD = 5 deg; with a random motion period). Next, children were given 8 trials of increasing difficulty. Trial-by-trial feedback on accuracy was provided in the demonstration, practice and criterion trials (unlike in the experimental phase), as well as ‘timeout’ messages if no response was made within 2500 msec (‘That was correct!’, ‘It was the other way that time’, ‘Timeout! Try to be quicker next time!’). In the experimental phase of each task, two randomly interleaved difficulty conditions were presented. In the motion coherence task, the levels of difficulty were 75% coherence (“easy”) and 30% coherence (“difficult”). In the direction integration task, the difficulty levels corresponded to standard deviations of directions of 30 deg (“easy”) and 70 deg (“difficult”). These difficulty levels were selected based on piloting and optimised for EEG, based on larger studies of drift-diffusion modelling in the context of perceptual decision-making (cf. Manning et al., 2021). We presented 72 trials for each difficulty condition (half of which contained leftward motion and half of which contained rightward motion). Additionally, we presented 8 randomly interleaved catch trials in each task where all dots moved either to the left or right (i.e., 100% coherence; SD = 0 deg) to provide reminders of the cue and ensure that children did not lose motivation. The trials in the experimental phase were divided into 4 blocks (corresponding to 4 ‘levels’ in each ‘game’ session), each consisting of 38 trials. Children did not have trial-by-trial feedback on their accuracy, but they were given ‘timeout’ feedback if they did not respond within 2500 msec. Furthermore, at the end of each block or ‘level’, children were given a score that reflected both their response time and accuracy ((1/median response time) ∗ number of correct responses ∗ 2, rounded to the nearest integer). The experimental code is available at https://osf.io/wmtpx/.

### 2.5 General procedure

The procedure of this study was approved by the local research ethics committee board. Written informed consent forms and assent forms were collected from parents and children, respectively, prior to participation. At the beginning of the EEG session, the sensor net was placed on the child's head and electrode impedances were checked; if necessary, adjustments were made to ensure that these were below 50 kΩ. EEG data were acquired at a sampling rate of 500 Hz with a vertex reference electrode. The child sat 80 cm away from the computer screen in a dimly-lit, electrically shielded room. A researcher sitting beside them provided general encouragement and task reminders, pausing before the start of a new trial where necessary (e.g., to remind the child to keep still or to not speak during the task). A short break was given at the end of each level, and a longer break was given between the two tasks. At this point, a new EEG recording session was started and children were offered some refreshments while electrode impedances were checked again to make sure they were below 50 kΩ, providing adjustments if required. To engage children in the ‘game’, they put a stamp on a record card after passing each ‘level’. The entire EEG session took approximately 1 h. In further sessions, children completed a Snellen acuity test, the Phonemic Decoding Efficiency subtest of the TOWRE-2 (Torgesen et al., 2012), the WIAT-III spelling (Wechsler, 2017), the WASI-2 (Wechsler, 2011) and the ADOS-2 (for autistic children only; Lord et al., 2012). Children were given a £10 voucher to thank them for their participation.

### 2.6 EEG data pre-processing

The pre-processing steps used by Manning et al. (2019) were followed. First, EEG data were band-pass filtered offline between .3 and 40 Hz using NetStation's filters before being exported as a binary file for further pre-processing in MATLAB. At this point, data were epoched into trials ranging from the fixation period onset to the end of the offset period and then median-corrected for DC offsets. Afterwards, bad electrodes were identified across each task's recording session, defined as those that have 15% or more samples exceeding the 97.5th absolute amplitude percentile for each participant, and replaced with the average of the nearest neighbouring electrodes (motion coherence task: M = 1.27% electrodes replaced per participant, range = 0–5.47%; direction integration task: M = 1.29% electrodes replaced per participant, range = 0–5.47%). We linearly regressed out the horizontal and vertical electrooculogram (EOG) from each channel. The horizontal EOG was calculated as the difference between the electrodes in the right and left outer canthi (electrodes 125 and 128) and the vertical EOG was calculated as the difference between the sum of electrodes positioned above the eyes (electrodes 8 and 25) and the sum of those placed on the cheeks (126 and 127). We removed channels on a trial-by-trial basis if they contained 15% or more samples exceeding the 97.5th absolute amplitude for each participant (motion coherence task: M = 3.89% data removed per participant, range = 1.06–7.32%; direction integration task: M = 3.78% data removed per participant, range = 1.09–6.55%). Next we substituted transients (samples that were four or more standard deviations away from the mean) with missing values. We removed EEG data from trials in which more than 15% of channels were removed (motion coherence task: M = 5.25% trials removed per participant, range = 0–16.45%; direction integration task: M = 5.01% trials removed per participant, range = 0–17.76%). We also removed the data from three electrodes for two participants which had no signal (the activity was flat). Finally, we converted the data to the average reference and baselined them to the average of the last 100 msec of the random motion period. Preprocessing scripts can be found at: https://osf.io/wmtpx/.

### 2.7 EEG analysis

Following Manning et al. (2019), we used a dimensionality reduction technique – reliable components analysis (RCA; Dmochowski et al., 2012; Dmochowski et al., 2014; Dmochowski & Norcia, 2015) – to identify components that maximised spatiotemporal trial-to-trial reliability. This method computes sets of electrode weights for each component, like principal components analysis (PCA). However, PCA components maximise variance explained, while RCA components maximise trial-to-trial covariance of the EEG. The trial-to-trial covariance criterion is appropriate for studying evoked responses as components of interest are expected to be spatiotemporally reproducible across trials, which is why grand averages across trials are often presented in standard event-related potential research. A forward-model projection of the weights can be used to visualise components as scalp topographies (Haufe et al., 2014; Parra et al., 2005), and data projected through these weights can be averaged for each timepoint to provide a time course for the component which can be compared across groups and conditions. Unlike traditional event-related potential analysis, our data-driven approach identifies topographic regions of interest using the whole electrode array while increasing the signal-to-noise ratio as each component represents a weighted average of electrodes. As a result, the approach also minimises experimenter degrees of freedom associated with selecting which electrodes to analyse. Despite this different approach, RCA yields components with timecourses that often reflect traditional event-related potential components (Dmochowski & Norcia, 2015; Manning et al., 2019).

We selected trial data from 100 msec prior to the stimulus onset to 600 msec following the stimulus onset. We applied RCA to the stimulus-locked data for the typically developing group for each task separately, to derive sets of normative component weights from which the performance of autistic and dyslexic groups could then be compared. For both tasks, the two most reliable components resembled those reported by Manning et al. (2019). The first most reliable component was maximal over centro-parietal electrodes, and the second most reliable component was maximal over occipital electrodes. Together the first two components explained 56.5% of the total trial-by-trial reliability in typically developing participants in the motion coherence task and 53.9% of the total reliability in the direction integration task. Our preregistered hypotheses were focused on the second most reliable component (component 2), although we also present exploratory analyses for component 1.

We then projected each group's data through the component weights derived for the typically developing children and averaged these to provide component waveforms. This approach allowed us to directly compare the response dynamics for each component across the groups, and characterise the extent to which the responses in the autistic and dyslexic groups deviated from the typically developing group. However, we also obtained the same pattern of results when projecting the data through weights derived from all participants together in an exploratory analysis (see Supplementary Figures S1 and S2). As in Manning et al. (2019), we projected a longer record of stimulus-locked data through the weights (from −100 msec before stimulus onset to 800 msec after stimulus onset), to more extensively characterise the temporal evolution of the components. We also conducted exploratory analyses on response-locked data from −600 msec before the response to 200 msec after the response (for trials in which a behavioural response was made within 2500 msec), using response-locked weights obtained by RCA.

For each task, we assessed the effects of group and stimulus difficulty on the reliable component average waveforms with a mass univariate approach, using the second-level analysis functions from the LIMO EEG toolbox (Pernet et al., 2011). This approach allowed us to assess effects at each timepoint, while using a temporal clustering technique to control for multiple comparisons (see Maris, 2012; Groppe et al., 2011 and Pernet et al., 2015, for review), as opposed to conducting statistical analyses on point measures of peak amplitude and latency. Importantly, this approach requires no *a priori* knowledge of precisely when an effect will occur and avoids difficulties with precisely determining the onset and offset of effects (Groppe et al., 2011). First, we centered the data for each group and coherence condition separately. Then, for each of 2000 bootstrap iterations, we randomly sampled with replacement the participants’ centered data and conducted a two-way (2 × 3) ANOVA with coherence condition as a repeated measures factor and group as a between-participants factor, in order to get a distribution of *F* values expected under the null hypothesis (i.e., 2000 *F* values for each factor/interaction at each timepoint; Pernet et al., 2015). We then used cluster statistics to control the family-wise error rate (Maris & Oostenveld, 2007; Pernet et al., 2015). We clustered the significant (*p* < .05) bootstrapped *F* values for each factor/interaction and used the maximum sum across clusters to derive a temporal cluster threshold for each factor/interaction with an alpha level of .05. Finally, we computed sums of temporal clusters of significant *F* values in the original, non-bootstrapped data and identified clusters that exceeded the cluster threshold. Where we obtained significant group effects, we conducted further two-way (2 × 2) ANOVAs at each timepoint within the cluster to compare typically developing children with autistic and dyslexic children, separately.

## 3 Results

### 3.1 Behavioural results

**Fig. 2:**
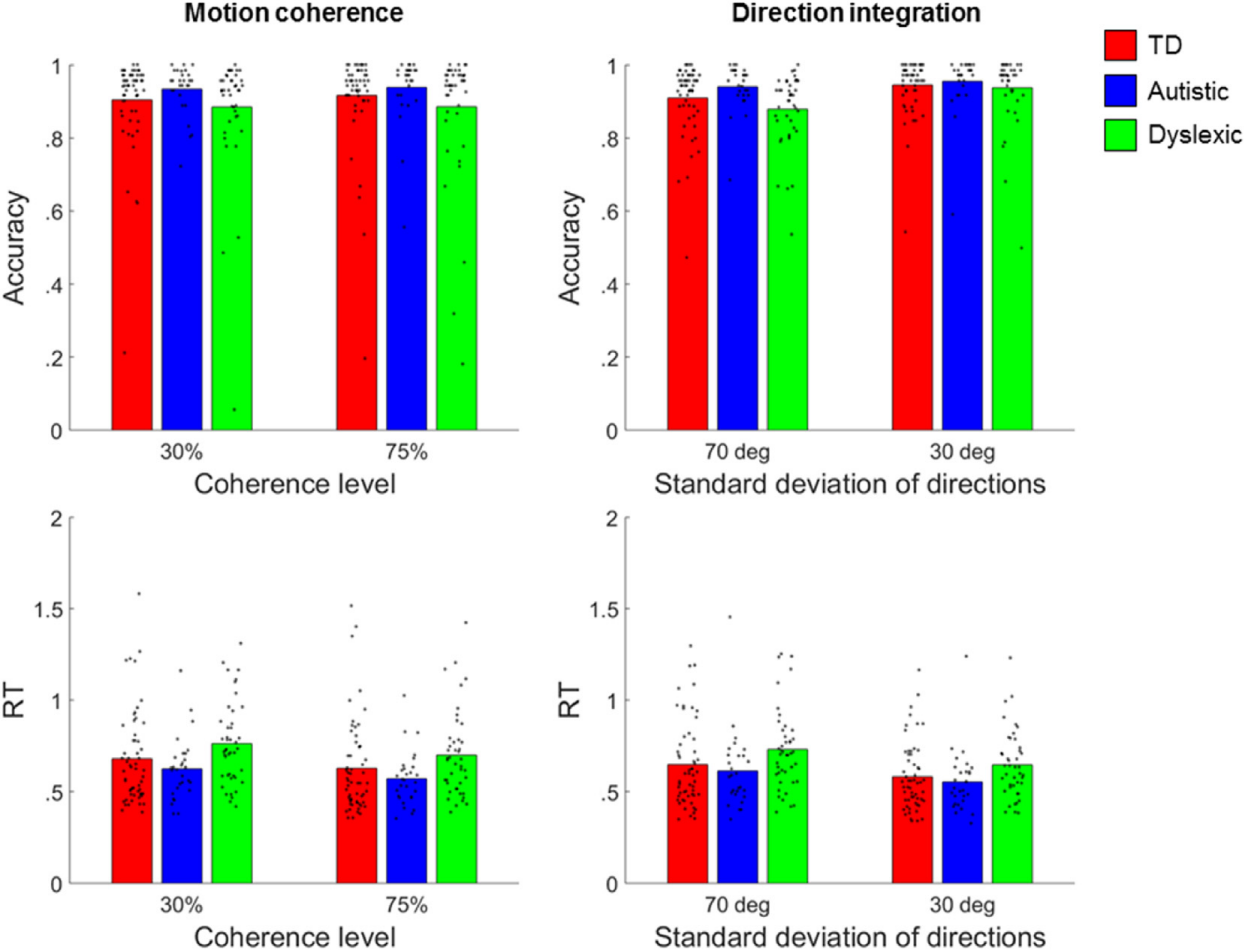
**Accuracy and response times for each group for the motion coherence (left) and direction integration (right) tasks.** Bars represent the mean accuracy (upper panels) and mean of median response times for correct trials (lower panels) for typically developing (TD; red), autistic (blue) and dyslexic (green) children. Dots represent the accuracy and median response time for correct trials for individual participants. Figure reproduced from https://osf.io/wmtpx/ under a CC-BY4.0 license.

Fig. 2 shows each group's mean accuracy and mean of median response times for correct trials in each task, along with individual data points. We investigated group differences in accuracy and median response times of correct trials using repeated-measures ANOVAs in IBM SPSS Statistics (Version 25) with difficulty level as a within-participants factor and group as a between-participants factor. For accuracy in the motion coherence task, there were no significant effects of group, *F*(2,127) = 1.29, *p* = .28, *η*_p_^2^ = .02, nor difficulty level, *F*(1,127) = 1.24, *p* = .27, *η*_p_^2^ = .01, nor a significant interaction between difficulty level and group, *F*(2,127) = .51, *p* = .60, *η*_p_^2^ < .01. However, there was a significant group effect on response time in the motion coherence task, *F*(2,127) = 3.27, *p* = .04, *η*_p_^2^ = .05. Planned simple contrasts showed no significant differences between autistic and typically developing children, *p* = .28, nor between dyslexic and typically developing children, *p* = .09, although visual inspection of Fig. 2 shows that, on average, dyslexic children were slightly slower and autistic dyslexic children slightly faster than typically developing children. There was also a significant effect of difficulty level on response times, *F*(1,127) = 78.76, *p* < .001, *η*_p_^2^ = .38, with slower response times in the most difficult condition, as expected. The interaction between difficulty level and group was not significant, *F*(2, 127) = .34, *p* = .71, *η*_p_^2^ < .01.

In the direction integration task, there was no significant effect of group on accuracy, *F*(2,124) = 1.86, *p* = .16, *η*_p_^2^ = .03, but there was a significant effect of difficulty level, *F*(1,124) = 73.59, *p* < .001, *η*_p_^2^ = .37, and an interaction between group and difficulty level, *F*(2,124) = 8.14, *p* < .001, *η*_p_^2^ = .12. A one-way ANOVA for each difficulty level found significant group differences in accuracy only in the difficult condition, *F*(2,124) = 3.91, *p* = .02, *η*_p_^2^ = .06, and not the easy condition, *F*(2,124) = .37, *p* = .69, *η*_p_^2^ < .01. Planned simple contrasts for the difficult condition revealed no significant differences between autistic and typically developing children, *p* = .15, or between dyslexic and typically developing children, *p* = .10. Although Fig. 2 shows that dyslexic children had a slightly higher mean of median response times compared to typically developing and autistic children, as in the motion coherence task, we found no overall effect of group on response times in this task, *F*(2,124), = 2.75, *p* = .07, *η*_p_^2^ = .04, and no interaction between group and difficulty level, *F*(2,124) = .98, *p* = .38, *η*_p_^2^ = .02.

### 3.2 Do autistic children and dyslexic children exhibit a reduced N2-like peak during the motion coherence task compared to typically developing children?

**Fig. 3:**
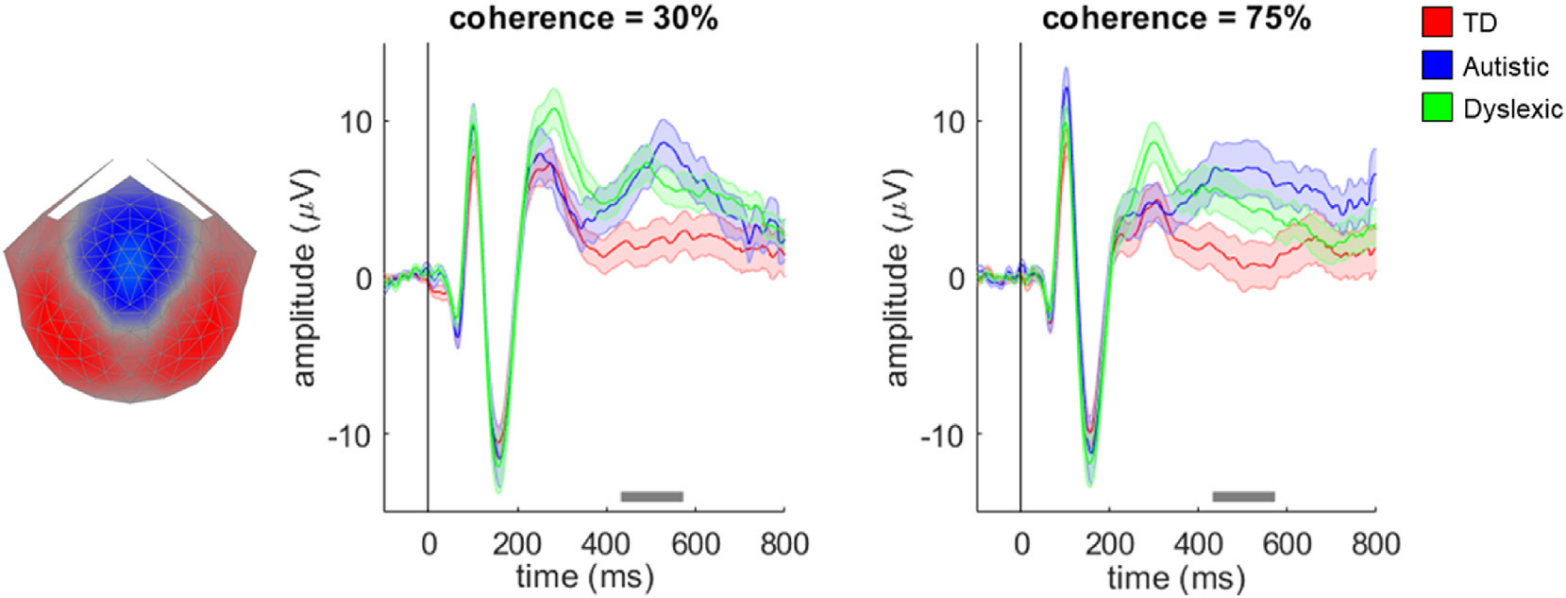
**Scalp topography for stimulus-locked component 2 and average waveforms for each group in the motion coherence task.** Topographic visualisation of the forward-model projection of component 2 reflecting the weights given to each electrode following reliable components analysis (RCA) on stimulus-locked data from the typically developing group in the motion coherence task, pooled across difficulty levels (left panel). The waveforms show the data from each group (red: typically developing (TD); blue: autistic; green: dyslexic) multiplied by the electrode weights, for the ‘difficult’ condition (coherence = 30%, central panel) and the ‘easy’ condition (coherence = 75%, right panel). Shaded error bars represent the standard error of the mean. The grey horizontal bars represent a cluster-level effect of group (main effect) between 432 and 572 msec. Figure reproduced from https://osf.io/wmtpx/ under a CC-BY4.0 license.

To address our first hypothesis, we multiplied the data from each individual in the motion coherence task by the component 2 electrode weights obtained from RCA for the typically developing group and averaged them together to form a single waveform for each participant. The grand average waveforms for each group shown in Fig. 3 follow a similar pattern of four initial peaks – first there is a small negative peak at ~60 msec, followed by a large positive peak at ~100 msec (a P1-like peak), a large negative peak at ~160 msec (a N2-like peak) and then another large positive peak at ~240–300 msec (a P2-like peak). The topographical distributions of activity corresponding to each peak are presented in Supplementary Figure S3. In a previous study of typically developing children (Manning et al., 2019), the small initial negative peak was not apparent and the P1-like and N2-like peaks were slightly later. These differences could be due to the addition of an auditory cue highlighting stimulus onset in the current study.

The mass univariate approach revealed a significant within-participants effect of difficulty level, with clusters between 210 msec and 298 msec, and between 484 msec and 598 msec. However, we were most interested in the between-participants effect of group, for which there was a significant effect corresponding to a cluster between 432 msec and 572 msec. To understand the source of these group differences, we conducted separate analyses within the cluster to compare autistic and typically developing children and dyslexic and typically developing children. Both autistic and dyslexic children had significantly higher amplitudes than typically developing children. This corresponded to a cluster extending from 434 msec to 572 msec in the analysis comparing autistic and typically developing children, and a cluster extending from 432 msec to 518 msec in the analysis comparing dyslexic and typically developing children. There was no significant interaction between group and difficulty level. In contrast to our hypothesis, the N2-like peak appeared to be of comparable amplitude across groups, with group differences emerging considerably later than the N2-like peak.

### 3.3 Do autistic and dyslexic children differ from typically developing children in N2-like peak amplitude during a direction integration task?

**Fig. 4:**
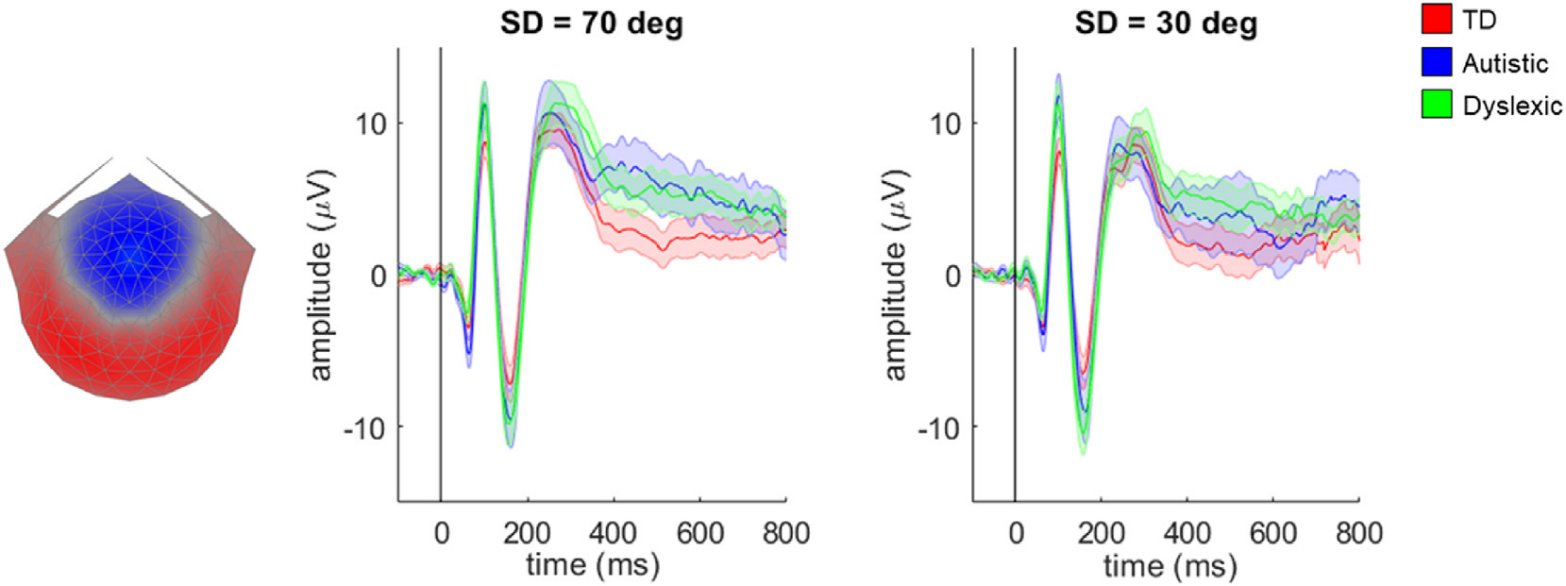
**Scalp topography for stimulus-locked component 2 and average waveforms for each group in the direction integration task.** Topographic visualisation of the forward-model projection of component 2 reflecting the weights given to each electrode following reliable components analysis (RCA) on stimulus-locked data from the typically developing group in the direction integration task, pooled across difficulty levels (left panel). The waveforms show the data from each group (red: typically developing (TD); blue: autistic; green: dyslexic) multiplied by the electrode weights, for the ‘difficult’ condition (SD of dot directions = 70 deg, central panel) and the ‘easy’ condition (SD of dot directions = 30 deg, right panel). Shaded error bars represent the standard error of the mean. Figure reproduced from https://osf.io/wmtpx/ under a CC-BY4.0 license.

To address our second hypothesis, we applied the same approach to the data from the direction integration task. As in the motion coherence task, the grand average waveforms for each group in Fig. 4 also had a small, initial negative peak, followed by large P1-like, N2-like and P2-like peaks, at ~100 msec, ~160 msec, and ~240–300 msec, respectively. Supplementary Figure S4 shows the topographical plots corresponding to each of these peaks. In this task, there was no significant between-participants effect of group, nor interaction between difficulty level and group on component average waveforms. The only significant effect was for difficulty level, corresponding to a cluster between 208 msec and 496 msec. Therefore, we did not find evidence in support of our hypothesis that N2-like peak amplitudes vary between groups.

### 3.4 Exploratory analyses: individual differences in N2-like peak amplitude

Contrary to our hypotheses, we found no significant group differences in N2-like peak in either task. Yet, there was considerable within-participants variability in behavioural performance (Fig. 2) and evoked potentials (see Supplementary Figures S5 and S6 for individual component waveforms; see also Kubová et al., 2014: although we note that the current waveforms were obtained over 72 trials per condition, *vs* 40 in Kubová et al., 2014 potentially leading to sharper peaks). We therefore conducted exploratory analyses to investigate whether individual N2-like peak amplitudes were related to behavioural performance and/or participant characteristics. We found each individual's N2-like peak amplitude by finding the minimum amplitude in their average component 2 waveform for each difficulty level between 100 msec and 250 msec, corresponding to the positive peaks either side of the N2-like peak found in the group average waveforms (see also Martin et al., 2010). The average peak amplitudes for each group are provided in Supplementary Table 1. We then averaged the N2-like peak amplitude across difficulty levels and conducted Pearson correlations between this measure and behavioural performance measures (accuracy and median RT for correct trials, after excluding trials which were excluded from EEG analyses) and participant characteristics (verbal IQ, performance IQ, SCQ, reading and spelling composite score). No correlations were significant in either the motion coherence task or the direction integration task (all *p* ≥ .15). Given the exploratory nature of these correlations, they will need to be further investigated in future work.

### 3.5 Exploratory analyses: N2-like peak latencies

While our pre-registered analyses focused on N2-like peak amplitude, we also looked at whether N2-like peak latencies might vary between groups, by comparing the latencies corresponding to the minimum amplitude in each individual's component 2 average waveform between 100 msec and 250 msec (see Supplementary Table 1 for group averages). Unexpectedly (cf. Kubová et al., 2015), there was some evidence that dyslexic children had slightly faster latencies than typically developing children specifically in the easy condition of the motion coherence task (Supplementary Figure S7).

### 3.6 Exploratory analyses: standard motion onset visual evoked potential measure

**Fig. 5:**
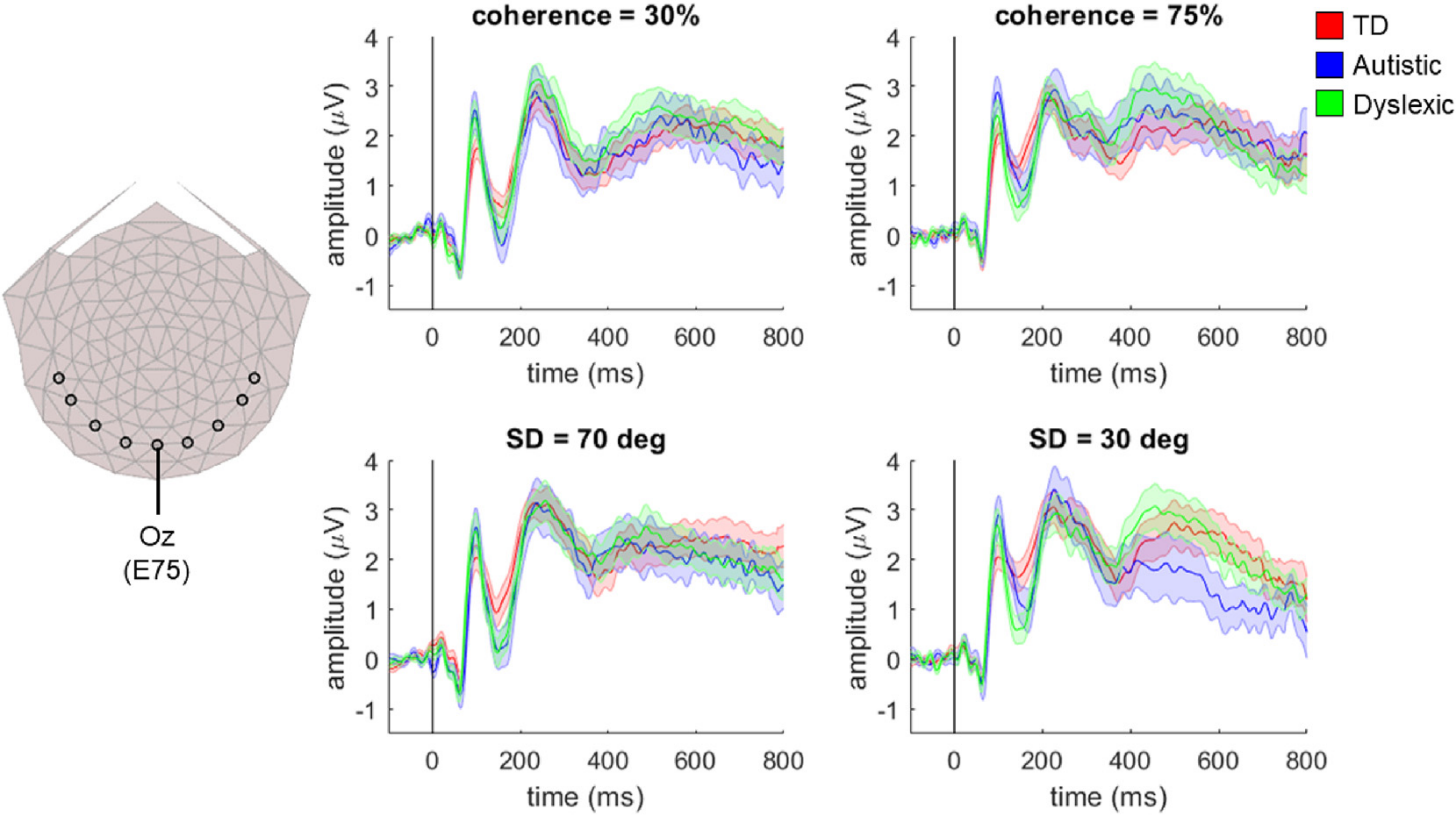
**Average waveforms across 9 occipital electrodes in the motion coherence and direction integration tasks.** The left panel shows the selected occipital electrodes (black circles; from left-to-right: E50, E58, E65, E70, E75 (Oz), E83, E90, E96, E101) from which activity was averaged to provide group average waveforms (right panels). The waveforms show the data from each group (red: typically developing (TD); blue: autistic; green: dyslexic) averaged across occipital electrodes, for the ‘difficult’ conditions (30% coherence in the motion coherence task, and SD = 70 deg in the direction integration task; central panel) and the ‘easy’ conditions (75% coherence in the motion coherence task, and SD = 30 deg in the direction integration task; right panel). Shaded error bars represent the standard error of the mean. Figure reproduced from https://osf.io/wmtpx/ under a CC-BY4.0 license.

We found no significant group differences in an N2-like peak identified by reliable components analysis. However, other studies using a more traditional event-related potential approach have reported differences in N2 amplitude in autistic and dyslexic individuals compared to typically developing individuals (e.g., Greimel et al., 2013; Jednoróg et al., 2011). We therefore conducted a supplementary analysis with a more traditional approach. As in Manning et al. (2019), we averaged activity across electrode Oz (electrode 75) and the four laterally positioned electrodes on either side (50, 58, 65, 70, 83, 90, 96, 101) for each participant. These electrodes were chosen to be comparable to the electrodes used by Niedeggen and Wist (1999) to study coherence-onset visual evoked potentials. Group average waveforms for activity averaged across these 9 occipital electrodes are presented in Fig. 5. The same pattern of peaks can be seen here as in Fig. 3, Fig. 4, including our peak of interest: an N2 peak, at ~180 msec.

We again investigated effects at each timepoint with a 3 (group) by 2 (difficulty level) bootstrapped ANOVA for each task, using the mass univariate approach. For both tasks, there were significant within-participants effects of difficulty level (motion coherence task: clusters between 74 msec and 214 msec and between 304 msec and 488 msec; direction integration task: clusters between 114 msec and 206 msec and between 666 msec and 762 msec), but no significant group or interaction effects. Therefore, the results of this more traditional analysis show no evidence of differences in early time points including the N2 peak, in line with the results from our reliable components analysis technique. Interestingly, the significant group difference found at later time points in the motion coherence task using our reliable components technique (Fig. 3) was not found in this analysis, suggesting that group differences in the reliable component predominantly reflect activity beyond the medial occipital electrodes selected for this average of electrodes approach.

### 3.7 Exploratory analyses: decision-related activity

**Fig. 6:**
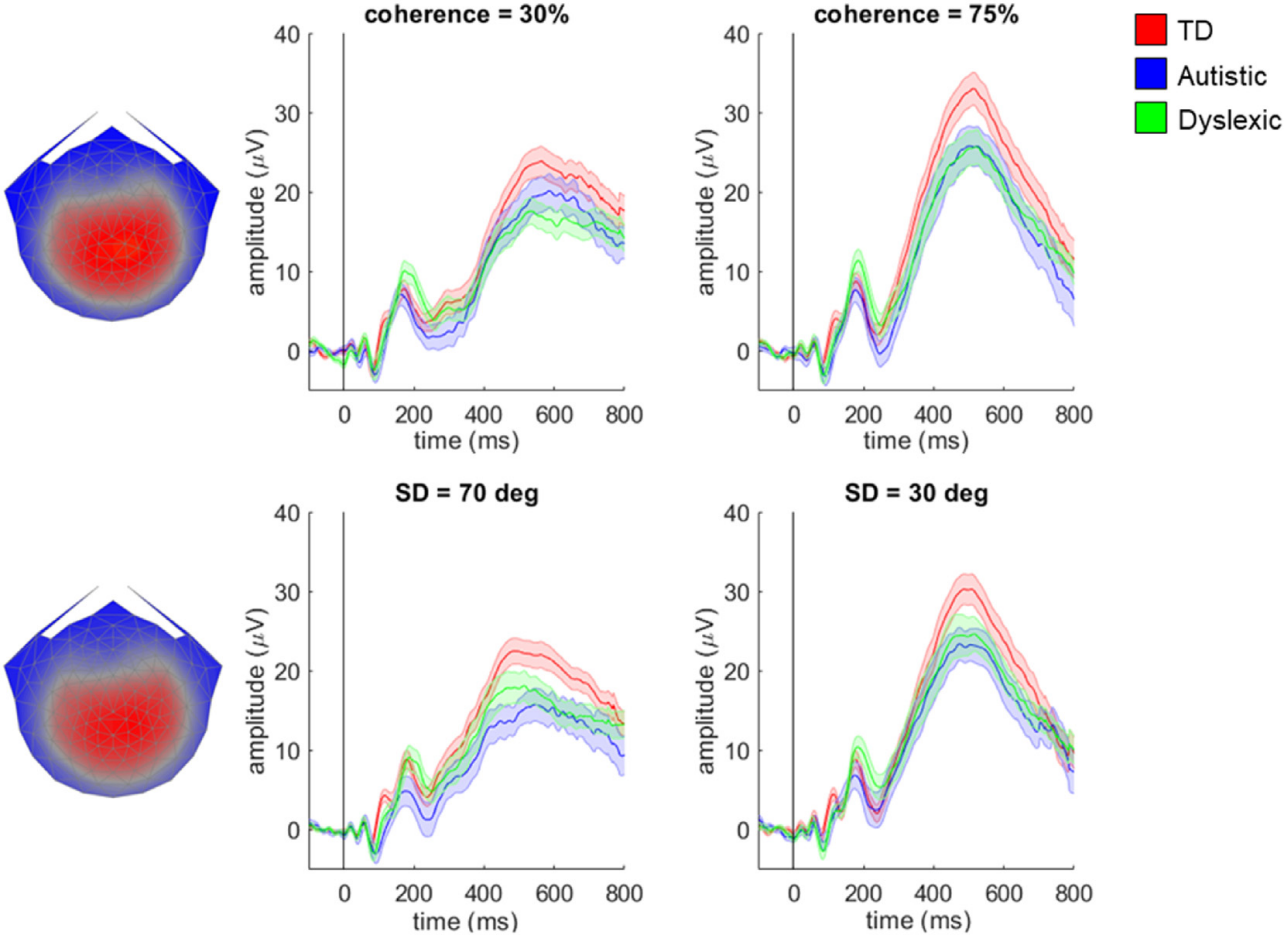
**Scalp topography for component 1 and average waveforms for each group in the motion coherence task (top) and direction integration task (bottom).** Topographic visualisation of the forward-model projection of stimulus-locked component 1 reflecting the weights given to each electrode following reliable components analysis (RCA) on data from the typically developing group in the motion coherence (top left) and direction integration tasks (bottom left), pooled across difficulty levels. The waveforms show the data from each group (red: typically developing (TD); blue: autistic; green: dyslexic) multiplied by the electrode weights, for the ‘difficult’ conditions (30% coherence in the motion coherence task, and SD = 70 deg in the direction integration task; central panel) and the ‘easy’ conditions (75% coherence in the motion coherence task, and SD = 30 deg in the direction integration task; right panel). Shaded error bars represent the standard error of the mean. Figure reproduced from https://osf.io/wmtpx/ under a CC-BY4.0 license.

Our pre-registered hypotheses focused on component 2, which was maximal over occipital electrodes, given the predominant focus of the published literature on N2. However, the most reliable component (component 1) resembled the stimulus-locked centro-parietal positivity purported to reflect decision-related activity (Kelly & O'Connell, 2013; see also Dmochowski & Norcia, 2015; Manning et al., 2019). Fig. 6 shows group average waveforms for component 1, which reflects the average stimulus-locked activity for each group multiplied by the electrode weights obtained from RCA analysis on the typically developing group. Unlike in our previous study of typically developing children (Manning et al., 2019), the component waveform shows a positive peak in all tasks at approximately 180 msec, which is likely attributable to the addition of the auditory cue to signal stimulus onset in this study. For this component we found significant effects of difficulty level for both tasks (corresponding to clusters between 294 msec and 622 msec and between 708 msec and 800 msec in the motion coherence task, and between 296 msec and 638 msec in the direction integration task). However there were no significant group or interaction effects.

**Fig. 7:**
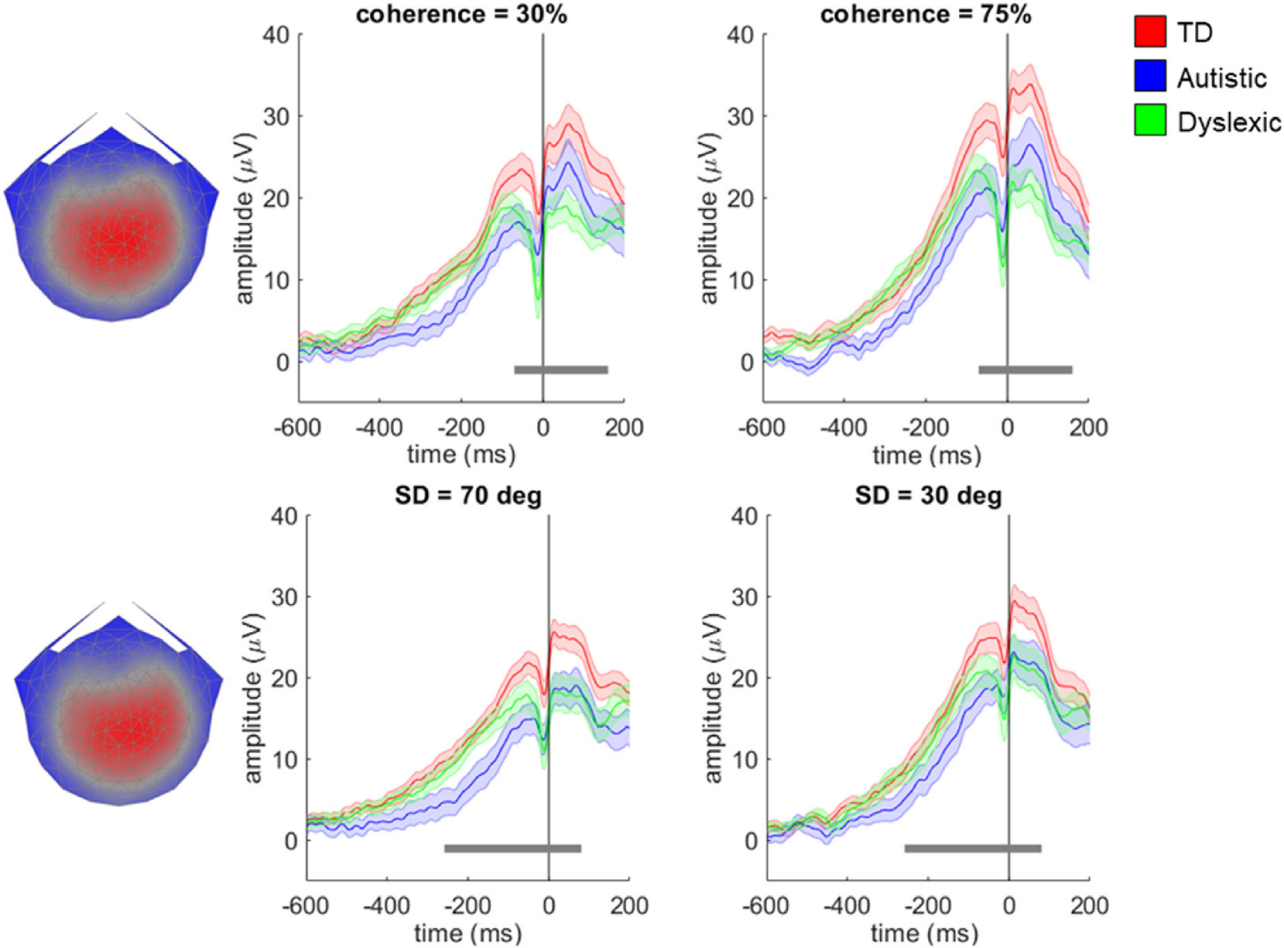
**Scalp topography for response-locked component 1 and average waveforms for each group in the motion coherence task (top) and direction integration task (bottom).** Topographic visualisation of the forward-model projection of response-locked component 1 reflecting the weights given to each electrode following reliable components analysis (RCA) on data from the typically developing group in the motion coherence (top left) and direction integration tasks (bottom left), pooled across difficulty levels. The waveforms show the data from each group (red: typically developing (TD); blue: autistic; green: dyslexic) multiplied by the electrode weights, for the ‘difficult’ conditions (30% coherence in the motion coherence task, and SD = 70 deg in the direction integration task; central panel) and the ‘easy’ conditions (75% coherence in the motion coherence task, and SD = 30 deg in the direction integration task; right panel). Shaded error bars represent the standard error of the mean. The grey horizontal bars represent a cluster-level effect of group (main effect) between −60 msec and 148 msec in the motion coherence task and between −254 msec and 76 msec in the direction integration task. Figure reproduced from https://osf.io/wmtpx/ under a CC-BY4.0 license.

**Fig. 8:**
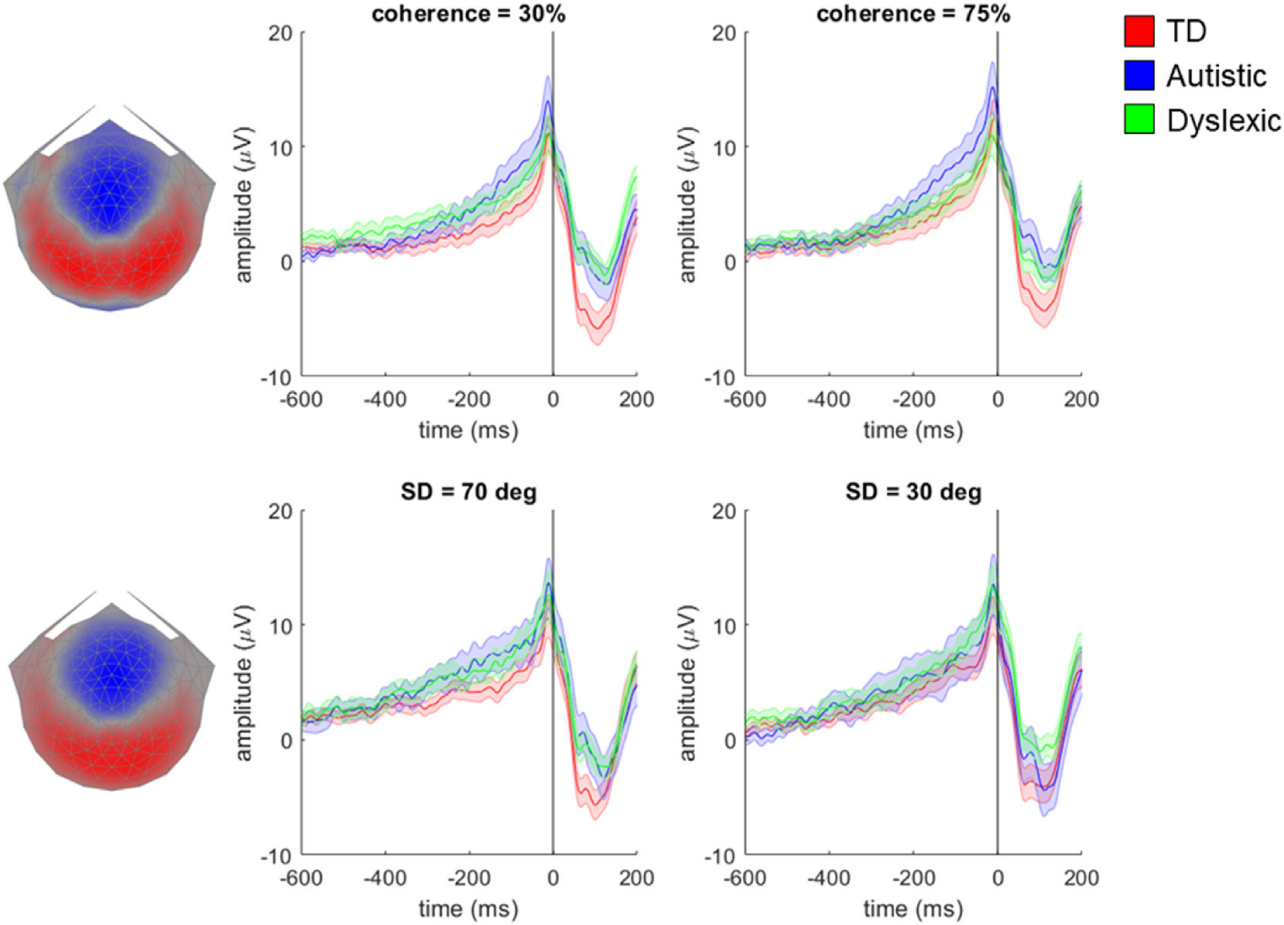
**Scalp topography for response-locked component 2 and average waveforms for each group in the motion coherence task (top) and direction integration task (bottom).** Topographic visualisation of the forward-model projection of response-locked component 2 reflecting the weights given to each electrode following reliable components analysis (RCA) on data from the typically developing group in the motion coherence (top left) and direction integration tasks (bottom left), pooled across difficulty levels. The waveforms show the data from each group (red: typically developing (TD); blue: autistic; green: dyslexic) multiplied by the electrode weights, for the ‘difficult’ conditions (30% coherence in the motion coherence task, and SD = 70 deg in the direction integration task; central panel) and the ‘easy’ conditions (75% coherence in the motion coherence task, and SD = 30 deg in the direction integration task; right panel). Shaded error bars represent the standard error of the mean. Figure reproduced from https://osf.io/wmtpx/ under a CC-BY4.0 license.

As decision-related activity is often analysed by assessing the data preceding the response (e.g., Kelly & O'Connell, 2013; Twomey et al., 2015) we also ran RCA on the typically developing group's data between −600 msec prior to the response and 200 msec after the response to obtain response-locked component weights. The topographical representations of the two most reliable response-locked components were similar to the stimulus-locked components for both tasks (see Fig. 7, Fig. 8). There was ramping activity preceding the response in both components, as reported in previous research in typical development (Manning et al., 2021). Fig. 7 presents each group's response-locked data multiplied by the weights for response-locked component 1. We found a main effect of group in both tasks, with a cluster between −70 msec and 160 msec in the motion coherence task, and between −258 msec and 80 msec in the direction integration task. Separate analyses comparing autistic children and typically developing children within these clusters revealed no significant differences in the motion coherence task, but a significant difference in the direction integration task. This difference corresponded to a cluster between −258 msec and −18 msec, with autistic children having lower amplitudes than typically developing children. Analyses comparing dyslexic children and typically developing children within these time windows revealed significant differences in both the motion coherence and direction integration tasks, corresponding to clusters between −60 and 160 msec and −30 msec and 80 msec, respectively, with dyslexic children having lower amplitudes than typically developing children. Therefore, it appears that autistic children have lower amplitudes than typically developing children in the direction integration task only, whereas dyslexic children have lower amplitudes than typically developing children in both tasks. There were also effects of difficulty condition in both tasks, corresponding to clusters from −200 msec to 94 msec in the motion coherence task, and from −172 msec to 104 msec in the direction integration task. There were no significant interaction effects in either task.

Fig. 8 presents each group's response-locked data multiplied by the weights for response-locked component 2. Here there were no main effects of group in either task and no interactions with difficulty level.

### 3.8 Exploratory analyses: better-matched groups

For our main analysis, we included all children who met our pre-registered inclusion criteria in order to prioritise representativeness and power, as opposed to following a strict group-matching approach. However, as shown in Table 1, the groups differed in age – with the dyslexic children having a higher minimum age than the children in the other groups, since dyslexia is only diagnosed after formal reading instruction has started. To create more closely age-matched groups, we selected only participants aged 8–14 years. We then projected the data from these subgroups through the RCA weights obtained from all of the typically developing children and re-ran the analyses for each task. Importantly, the same pattern of results was obtained in these parallel analyses: we did not see significant group differences in early evoked responses to motion even when the groups were more comparable in terms of age. In terms of response-locked activity, there were still significant group differences for component 1 in the motion coherence task, with dyslexic individuals having lower amplitudes than typically developing children near the time of the response. However, the group effect in the direction integration task was no longer significant.

## 4 Discussion

We used high-density EEG and a data-driven component decomposition technique to compare the motion-specific N2-like peak elicited by global motion onset in a motion coherence and direction integration task, in 57 typically developing, 29 autistic and 44 dyslexic children. Contrary to our hypotheses, we did not find any significant group differences in N2-like peak amplitude in either of our two tasks. However, in the motion coherence task we found significantly higher amplitudes in autistic and dyslexic children compared to typically developing children at later time points (around 430 msec–570 msec after stimulus onset), and found evidence of group differences in response-locked activity for both tasks in exploratory analyses.

First we consider the absence of significant group differences in N2-like peak amplitude between our groups. In line with Manning et al. (2019), we identified two main neural components with RCA. The second most reliable component was maximal over occipital electrodes and resembled previously reported coherent motion evoked potentials in both children and adults (Manning et al., 2019; Niedeggen & Wist, 1999; Patzwahl & Zanker, 2000). In all three groups and across both tasks, component 2 showed a small initial negativity at ~60 msec, followed by a large positivity at ~100 msec (P1-like peak), a large negativity at ~160 msec (N2-like peak) and another large positivity at ~240–400 msec (P2-like peak). While the P1 is not normally found in response to coherent motion onset following random motion in adult observers, we previously reported that the P1 was present in children and that its dominance reduces with age (Manning et al., 2019). This previous study did not however show an initial negativity at ~60 msec, and the latencies of the following peaks were generally longer – differences which may be attributable to the addition of an auditory tone at stimulus onset in the current study. Across both motion tasks, we found no evidence of amplitude differences in the N2-like peak, which has been proposed as a marker of motion-specific processing (Clifford & Ibbotson, 2003; Heinrich, 2007). We reached the same conclusion when using a more traditional event-related potential approach by averaging across occipital electrodes (e.g., Niedeggen & Wist, 1999), demonstrating that the failure to find group differences was not due to the data-driven RCA method used. Furthermore, we replicated these results in smaller groups that were comparable in age. Exploratory analyses suggested that N2-like peak latencies might be shorter for dyslexic children than typically developing children in the easy condition of the motion coherence task, but as these differences were not predicted *a priori* and were in the opposite pattern to previous results using motion-onset visual evoked potentials (Kubová et al., 2015), potential latency differences will need to be explored further in future research.

Our results contrast those of Greimel et al. (2013), who reported a reduced N2 peak amplitude amongst autistic children and adolescents in a motion coherence task similar to ours. The failure to replicate this finding could be due to differences in stimulus parameters (e.g., coherence levels, dot size and speed), and alerting effects brought about by the inclusion of the auditory tone to signal stimulus onset in the current study. However, we also note that Greimel et al. (2013) had a relatively small sample size (n = 16 autistic and n = 12 control participants), and the participants were slightly older (8–16 years) than those tested here. Our results are more consistent with previous studies of coherent motion evoked potentials in dyslexia, which do not find evidence of overall differences in N2 peak amplitude (Taroyan et al., 2011; Scheuerpflug et al., 2004; Jednoróg et al., 2011), albeit in tasks which did not present a period of random motion before coherent motion onset. To our knowledge, the only study to have used a task with a period of random motion before coherent motion onset did not report an N2 peak (Schulte-Körne et al., 2004). Notably, our study shows that these results extend to a novel direction integration task in which the standard deviation of dot directions is manipulated.

While we did not find evidence of differences in N2-like peak amplitudes, significant group differences emerged in component 2 around 430 msec–570 msec after stimulus onset, for the motion coherence task. Both the autistic and dyslexic children had higher amplitudes here compared to the typically developing children. Interestingly, Schulte-Körne et al. (2004) also suggested that differences between dyslexic and typically developing individuals were restricted to later processing stages following coherent motion onset (around 300 msec–800 msec), despite differences in analysis methods. We speculate that these differences might be linked to noise exclusion difficulties in autistic and dyslexic participants (see also Conlon et al., 2012; Manning et al., 2015; Sperling et al., 2005, 2006; Zaidel et al., 2015) as no group differences were found in the direction integration task, which does not require noise exclusion. Segregation in motion perception has been suggested to rely on feedback from higher-order areas (Raudies & Neumann, 2010), which could explain why group differences occur only around 430 msec after coherent motion onset. Along with previous behavioural studies (Manning et al., 2015, 2017), our study of neural dynamics in atypically developing populations reveals important differences in the nature of the two global motion tasks. We note that these differences at later timepoints in stimulus-locked component 2 were not apparent in a more traditional motion evoked potential method, showing the potential for RCA to provide additional insights.

In addition to our pre-registered analyses on our second most reliable RCA component (component 2), we investigated group differences in the most reliable stimulus-locked component (component 1), which resembled the decision-making variable reported in Manning et al. (2019). Here there were no significant group differences. However, response-locked activity – which is commonly used to assess decision-related processing (e.g., Kelly & O'Connell, 2013; Twomey et al., 2015) – revealed significant group differences preceding the response and extending past the response in component 1 (resembling the response-locked centro-parietal positivity; O'Connell et al., 2012; Kelly & O'Connell, 2013). The autistic children appeared to have significantly lower amplitudes than typically developing children in the direction integration task only, whereas dyslexic children had lower amplitudes than typically developing children in both tasks. These group differences could reflect differences in sustained responses to global motion, metacognitive processes (e.g., confidence; Herding et al., 2019; Murphy et al., 2015), and/or response-generation in autism and dyslexia. Interestingly, our results suggest that the two conditions may differ in these domains because of their differences across motion tasks. We note that group differences in response-locked component 1 were no longer present in the direction integration task when repeating the analysis on better age-matched groups, so future studies may benefit from investigating age-related changes within the groups.

We did not find clear behavioural differences between autistic and typically developing children, or between dyslexic and typically developing children, although the autistic children were slightly faster and more accurate, while the dyslexic children were slightly slower and less accurate. The behavioural results for the autism group stand in contrast to studies reporting reduced performance in motion coherence tasks and increased performance in the direction integration task (but note that not all studies have reported group differences; see van der Hallen et al., 2019 for review). The behavioural performance of the dyslexic children in the current study is in line with reports of elevated motion coherence thresholds in dyslexia (Benassi et al., 2010), although the group differences are slight. While there are many differences in task and stimulus parameters between this study and previous studies, we note that previous studies have generally measured psychophysical thresholds using a wide range of difficulty levels, whereas the current study only used two difficulty levels in each task, which were above threshold for most children. The difficulty levels used in the current study were chosen to elicit clear global motion onset evoked potentials and to enable modelling of the behavioural responses in future studies (Manning et al., 2021). We therefore do not wish to overemphasise the presence or absence of group differences in accuracy and response time in the current study in relation to previous studies assessing psychophysical thresholds: it is possible that group differences in behaviour may have become more pronounced for more difficult conditions. It is also interesting to consider whether group differences in N2-like peak amplitude may have emerged for more difficult conditions, although this is difficult to test as the N2 is not reliably elicited at low coherence levels (Niedeggen & Wist, 1999; Patzwahl & Zanker, 2000). It is also worth noting that Greimel et al. (2013) previously reported attenuated N2 amplitudes in autistic participants across a range of coherence levels (20%, 40% 60%) compared to typically developing children, despite no differences in behaviour.

Previously reported behavioural differences in motion processing in autistic and dyslexic individuals have been linked to atypical dorsal stream and/or magnocellular functioning (Braddick et al., 2003; Pellicano & Gibson, 2008; Spencer et al., 2000; Stein, 2001). Moreover, atypical evoked responses to coherent motion have been proposed to result from impaired magnocellular or dorsal stream functioning in these conditions (Greimel et al., 2013; Jednoróg et al., 2011; Schulte-Körne et al., 2004). In our analyses which assessed group differences at each timepoint, we found group differences only at relatively late stages of processing, ruling out accounts of generally impaired magnocellular or dorsal-stream functioning (see also Skottun & Skoyles, 2004; Skottun, 2011, for an argument against using coherent motion evoked potentials as a marker of magnocellular functioning). However, it is still possible that later processing stages may be affected specifically for motion processing, implicating later stages of the dorsal-stream pathway (e.g., parietal areas involved in decision-making; Kelly & O'Connell, 2013). Therefore, to further test the dorsal-stream hypothesis it will be important to compare evoked responses in motion tasks such as those presented here with well-equated form tasks to target the ventral stream.

Our results suggest that the nature of atypical motion processing may differ in autistic and dyslexic individuals. First, the two groups had different profiles of behavioural performance, with slightly reduced accuracy and increased response time found in the dyslexic group relative to the typically developing group, and the opposite pattern seen in the autistic group. Second, the autistic children differed in response-locked activity for the direction integration task only, whereas the dyslexic children differed in response-locked activity for both tasks. However, there also appeared to be shared characteristics, with both groups showing increased amplitudes in stimulus-locked activity in component 2 from approximately 430 msec. We note however that these results were not hypothesised, so future research will need to replicate these results. Furthermore, research linking these neural dynamics with behavioural performance is needed. The diffusion decision model (Ratcliff & McKoon, 2008) offers a possible framework to investigate neural dynamics relating to different aspects of the decision-making process. A further outstanding issue is the substantial individual variability in performance in all groups, and future research could aim to investigate the reasons for such variability.

In conclusion, in our pre-registered analyses we did not find evidence for differences in early stages of global motion processing including an N2-like peak in autism and dyslexia. However, we suggest that differences may arise at later processing stages reflecting sustained global motion responses, decision-making processes, metacognitive processes and/or response generation. Presenting two global motion tasks to autistic and dyslexic children allowed us to identify both areas of convergence and divergence between the two conditions in responses to motion. Such cross-syndrome approaches have the potential to progress our understanding of altered motion processing in different conditions. Future studies are needed to investigate atypicalities observed in later stages of motion processing in both autistic and dyslexic children, and ultimately, longitudinal studies will be required to determine whether they play a causally significant role in the development of these conditions.

## Data availability statement

We report how we determined our sample size, all data exclusions, all inclusion/exclusion criteria, whether inclusion/exclusion criteria were established prior to data analysis, all manipulations, and all measures in the study. In line with our ethics approval, parents/carers of participants consented to data being stored in a safeguarded repository so that other researchers could apply to use the data. The data can be accessed through the UK Data Service: https://dx.doi.org/10.5255/UKDA-SN-855018. Users will be required to register with the UK Data Service, sign the UK Data Service's End User License, and contact the corresponding author and provide their reason for wanting to access the data. Permission from the author is required as per the consent signed by participants. No further restrictions to data sharing will be applied other than what is included in the terms and conditions of the UK Data Service's End User License: https://ukdataservice.ac.uk/media/455131/cd137-enduserlicence.pdf. Code can be found here: https://osf.io/wmtpx/. Legal copyright restrictions prevent public archiving of the various assessment instruments described in the Participants section which can be obtained from the copyright holders in the cited references.

## Credit statement

**Lisa Toffoli:** Conceptualization, Data curation, Formal analysis, Investigation, Software, Writing – original draft. **Gaia Scerif:** Conceptualization, Funding acquisition, Methodology, Resources, Supervision, Writing – review & editing. **Margaret J. Snowling:** Conceptualization, Methodology, Writing – review & editing. **Anthony M. Norcia:** Conceptualization, Methodology, Writing – review & editing. **Catherine Manning:** Conceptualization, Data curation, Funding acquisition, Formal analysis, Investigation, Methodology, Software, Supervision, Writing – original draft.

## Footnotes

1 Note that one participant in the dyslexia group was currently in the process of obtaining a diagnosis. This participant was retained in the sample as their reading and spelling composite score was below 89.
