# Supplementary material for: Global motion evoked potentials in autistic and dyslexic children: A cross-syndrome approach
Source: Cortex. 2021 Oct;143:109–26. doi: 10.1016/j.cortex.2021.06.018 (PMC8500218; doi:10.1016/j.cortex.2021.06.018)
Supplement: Multimedia component 1 [file mmc1.docx]

**Supplementary Materials**

**Table S1. Summary statistics for N2-like peak amplitude extracted for each individual**

|  | TD | Autistic | Dyslexic |
| --- | --- | --- | --- |
| *Motion coherence task* | | | |
| 30% coherence: amplitude | -12.16 (8.12) | -13.26 (9.56) | -13.46 (11.60) |
| 75% coherence: amplitude | -11.55 (7.82) | -12.91 (10.42) | -13.61 (10.57) |
| 30% coherence: latency | 158.18 (11.69) | 158.90 (16.73) | 157.68 (12.62) |
| 75% coherence: latency | 162.84 (24.28) | 164.55 (27.23) | 151.86 (14.05) |
| *Direction integration task* | | | |
| 70 deg SD: amplitude | -8.91 (8.57) | -11.38 (9.85) | -11.42 (9.66) |
| 30 deg SD: amplitude | -8.44 (7.79) | -10.99 (10.30) | -11.75 (9.48) |
| 70 deg SD: latency | 162.43 (22.47) | 159.50 (23.48) | 158.00 (11.15) |
| 30 deg SD: latency | 163.86 (24.29) | 161.14 (11.98) | 156.23 (13.20) |

**Note.** Data are presented as mean (SD). N2-like peak was extracted as the minimum amplitude in each individual’s component 2 average waveform between 100 ms and 250 ms.


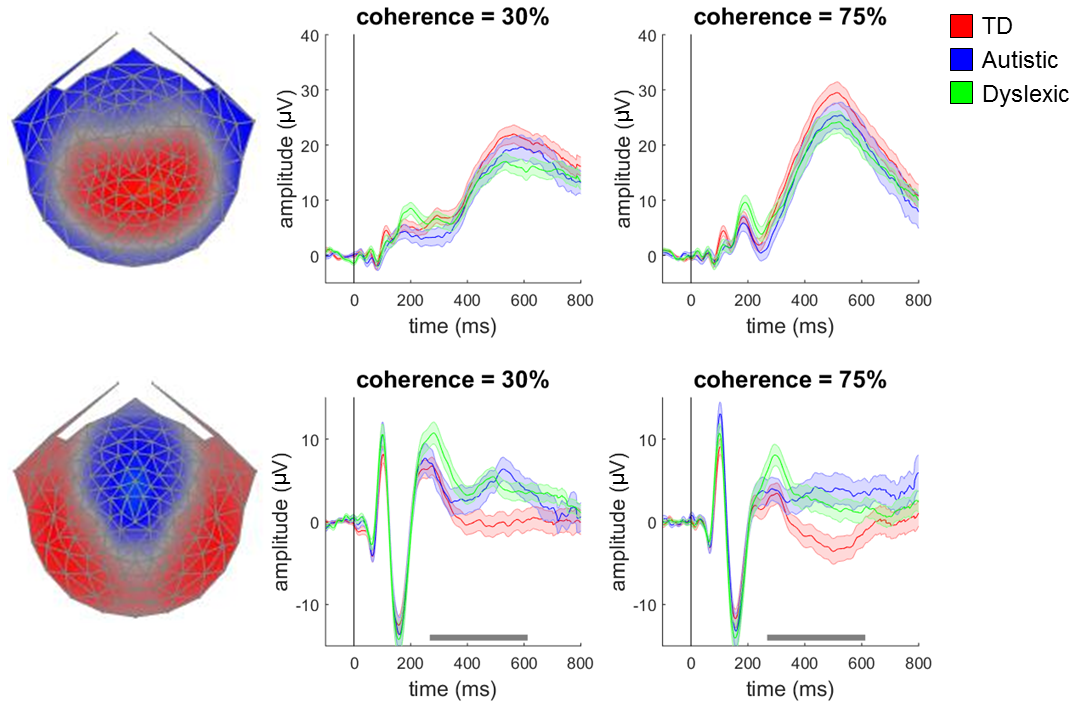


**Figure S1. Scalp topography of stimulus-locked electrode weights obtained from RCA on all participant’s data and average component waveforms for each group in the motion coherence task**

In the main analysis reported in the text, we multiplied each group’s data by weights obtained from reliable components analysis (RCA) on stimulus-locked data from the typically developing group. Here, we show the topographic visualisation of the forward-model projection of component 1 (top left) and component 2 (bottom left) for weights obtained from reliable components analysis (RCA) on data from all participants in the motion coherence task, pooled across difficulty levels. The waveforms show the data from each group (red: typically developing (TD); blue: autistic; green: dyslexic) multiplied by the electrode weights, for the ‘difficult’ condition (coherence = 30%, central panel) and the ‘easy’ condition (coherence = 75%, right panel). Shaded error bars represent the standard error of the mean. The grey horizontal bars represent a cluster-level effect of group (main effect) for the motion coherence task between 376 ms and 608 ms.


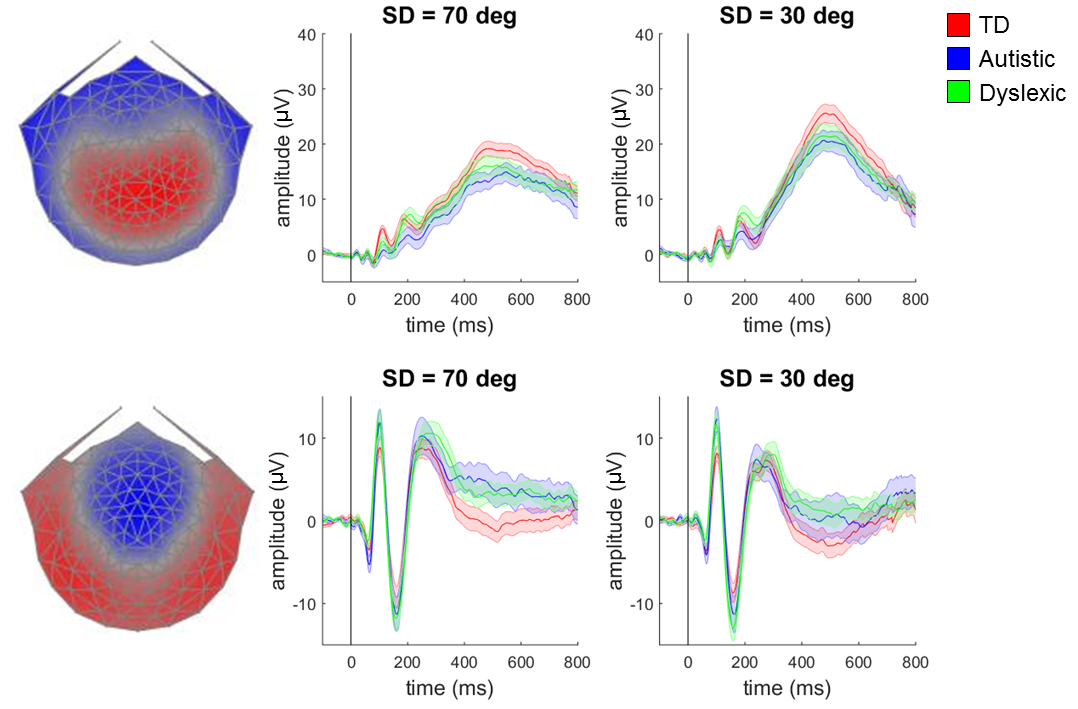


**Figure S2. Scalp topography of response-locked electrode weights obtained from RCA on all participant’s data and average component waveforms for each group in the direction integration task**

In the main analysis reported in the text, we multiplied each group’s data by weights obtained from reliable components analysis (RCA) on response-locked data from the typically developing group. Here, we show the topographic visualisation of the forward-model projection of component 1 (top left) and component 2 (bottom left) for weights obtained from reliable components analysis (RCA) on data from all participants in the direction integration task, pooled across difficulty levels. The waveforms show the data from each group (red: typically developing (TD); blue: autistic; green: dyslexic) multiplied by the electrode weights, for the ‘difficult’ condition (SD of dot directions = 70 deg; central panel) and the ‘easy’ condition (SD of dot directions = 30 deg; right panel). Shaded error bars represent the standard error of the mean.


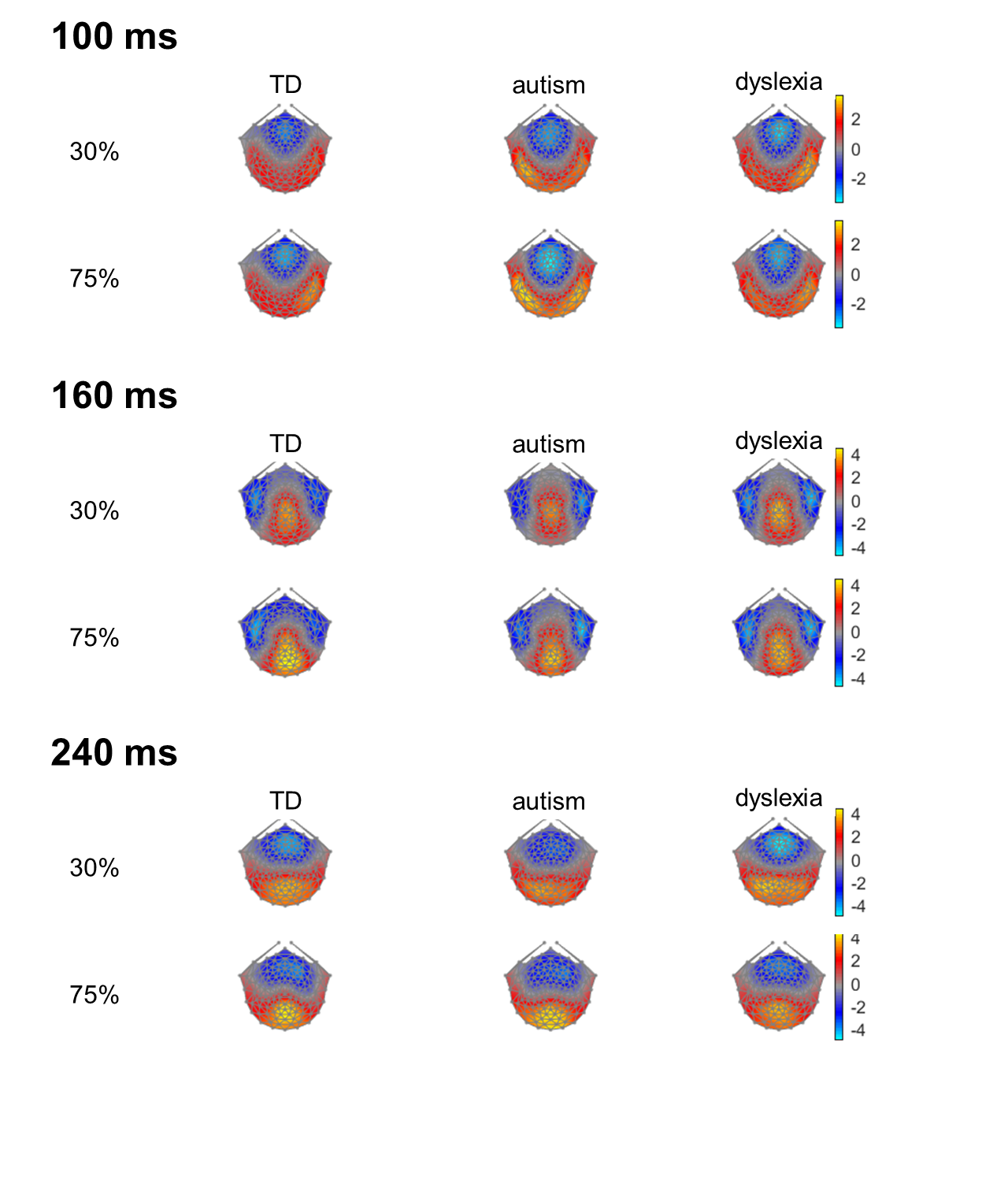


**Figure S3. Topographical distribution of activity at 100 ms, 160 ms and 240 ms after stimulus onset in the motion coherence task**

Topographical plots reflect activity averaged across typically developing (TD) children (left column), autistic children (middle column) and children with dyslexia (right column) at each timepoint (100 ms, 160 ms, and 240 ms). The timepoints chosen correspond to the component 2 peaks shown in Figure 3. Within each panel, the top row shows the topographical plots for the difficult (30% coherence) condition and the bottom row shows the topographical plots for the easy (75% coherence) condition.


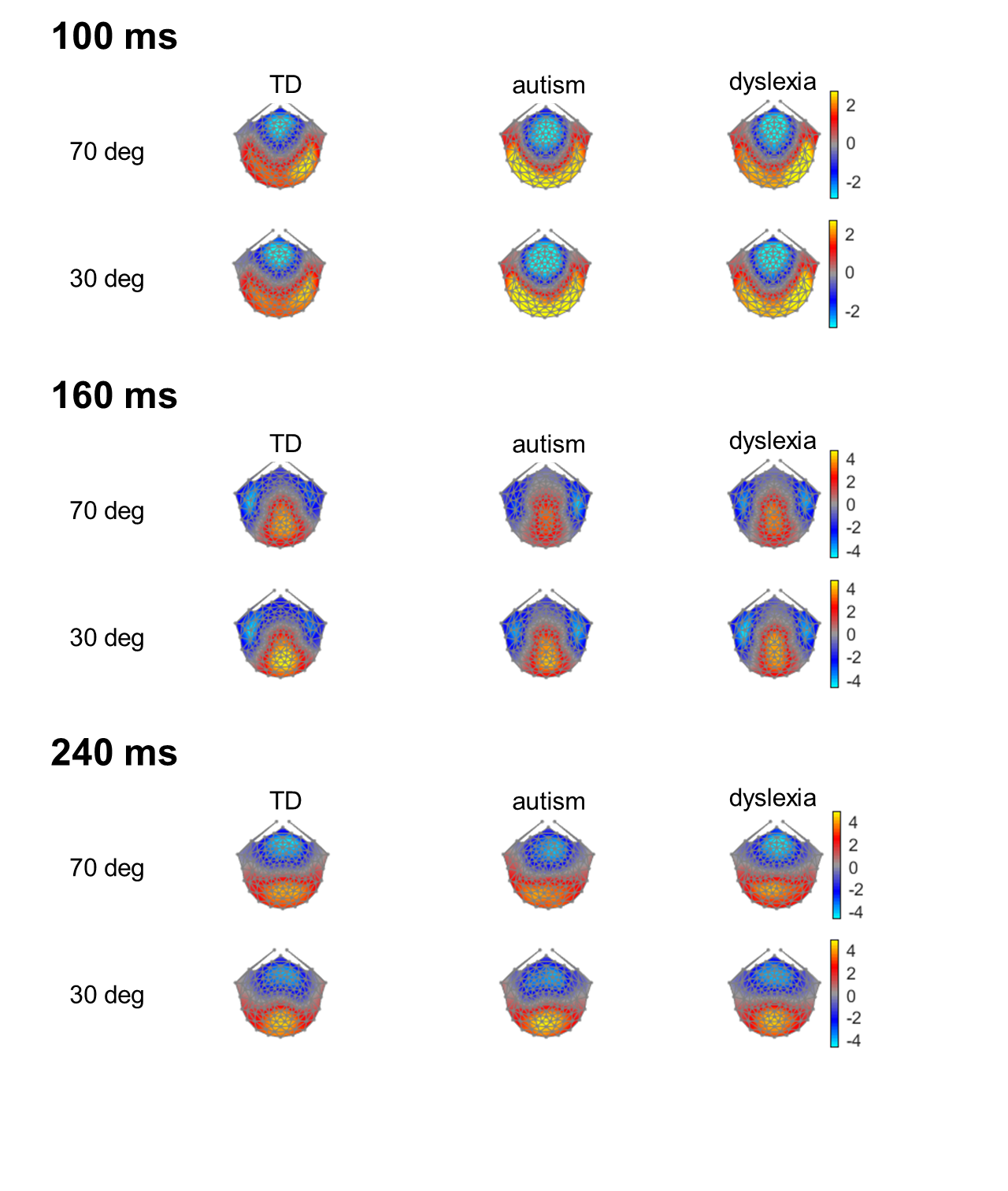


**Figure S4. Topographical distribution of activity at 100 ms, 160 ms and 240 ms after stimulus onset in the direction integration task**

Topographical plots reflect activity averaged across typically developing (TD) children (left column), autistic children (middle column) and children with dyslexia (right column) at each timepoint (100 ms, 160 ms, and 240 ms). The timepoints chosen correspond to the component 2 peaks shown in Figure 4. Within each panel, the top row shows the topographical plots for the difficult (SD = 70 deg) condition and the bottom row shows the topographical plots for the easy (SD = 30 deg) condition.


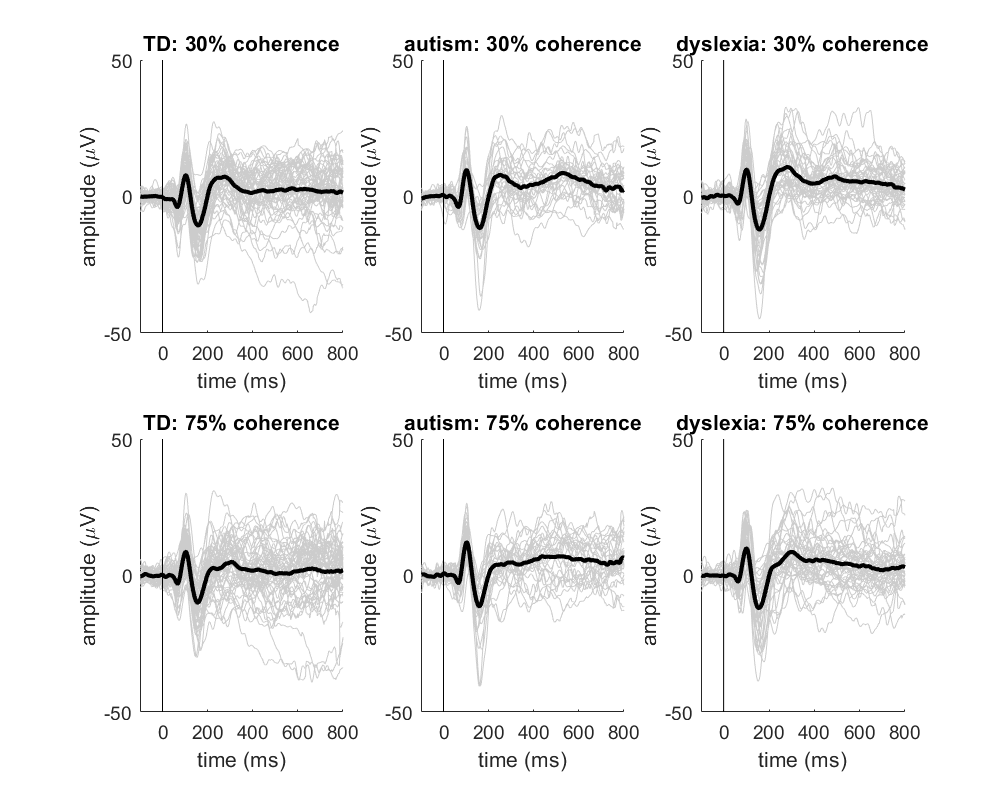


**Figure S5: Individual component 2 waveforms for each group in the motion coherence task**

Grey lines represent component 2 average waveforms for each participant in the typically developing group (TD; left), autism group (centre) and dyslexia group (right), for the ‘difficult’ condition (upper panels, 30% coherence) and for the ‘easy’ condition (lower panels, 75% coherence). These component waveforms were obtained by multiplying each individual’s data by the electrode weights that resulted from RCA on the typically developing group data. The thick black line represents the group average waveform.

**
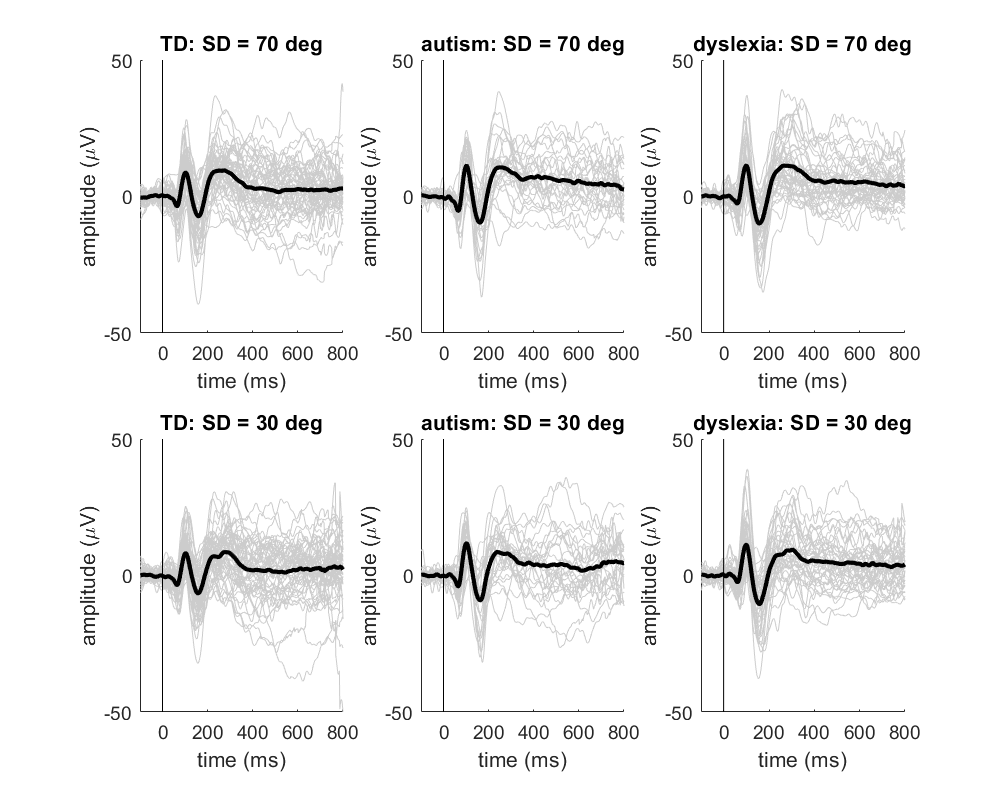
Figure S6: Individual component 2 waveforms for each group in the direction integration task**

Grey lines represent component 2 average waveforms for each participant in the typically developing group (TD; left), autism group (centre) and dyslexia group (right), for the ‘difficult’ condition (upper panels, SD of dot directions = 70 deg) and for the ‘easy’ condition (lower panels, SD of dot directions = 30 deg). These component waveforms were obtained by multiplying each individual’s data by the electrode weights that resulted from RCA on the typically developing group data. The thick black line represents the group average waveform.

**
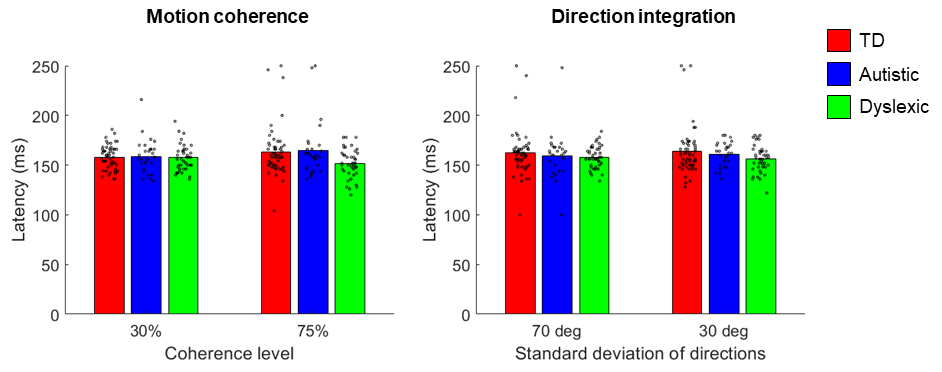
**

**Figure S7. N2-like peak latencies in component 2 for typically developing, autistic and dyslexic children for the motion coherence task (left) and direction integration task (right)**

Our pre-registered hypotheses concerned group differences in N2-like peak amplitude. However, we also investigated group differences in N2-like peak latency in exploratory analyses. We extracted a peak latency in component 2 for each participant corresponding to the minimum amplitude between 100 ms and 250 ms. We then compared peak latencies between groups in a 2 (difficulty level) x 3 (group) ANOVA for each task in IBM SPSS Statistics version 25. There was no significant main effect of group in either the motion coherence task [*F*(2,127) = 2.53, *p* = .08, *η*_p_^2^ = .04] or direction integration task [*F*(2,124) = 1.77, *p* = .18, *η*_p_^2^ = .03]. There was also no significant interaction between group and difficulty level in the direction integration task [*F*(2,124) = .32, *p* = .73, *η*_p_^2^ < .01]. However, the interaction was significant in the motion coherence task [*F*(2,127) = 3.88, *p* = .02, *η*_p_^2^ = .06]. Separate one-way ANOVAs for each condition revealed a significant difference between groups in the easiest condition only, with dyslexic children having reduced latencies compared to typically developing children, with no significant difference between latencies in autistic and typically developing children.
